# Isolation and Characterization of Antimicrobial Metabolites from the *Sophora tonkinensis*-Associated Fungus *Penicillium* sp. GDGJ-N37

**DOI:** 10.3390/molecules29020348

**Published:** 2024-01-10

**Authors:** Lili Huang, Yongxia Li, Jing Pang, Liuxia Lv, Jiatong Zhou, Liqi Liang, Xianhua He, Jun Li, Weifeng Xu, Ruiyun Yang

**Affiliations:** State Key Laboratory for Chemistry and Molecular Engineering of Medicinal Resources, School of Chemistry and Pharmaceutical Sciences, Guangxi Normal University, Guilin 541004, China; 17878190355@163.com (L.H.); 18177405482@163.com (Y.L.); pangjing20220806@163.com (J.P.); liuxialv1997@163.com (L.L.); zjt990123@163.com (J.Z.); 13471527876@163.com (L.L.); 15307733536@163.com (X.H.); lijun9593@gxnu.edu.cn (J.L.)

**Keywords:** *Sophora tonkinensis*, *Penicillium* sp., azaphilone derivatives, antibacterial activities, antifungal activities

## Abstract

Chemical investigation of *Penicillium* sp. GDGJ-N37, a *Sophora tonkinensis*-associated fungus, yielded two new azaphilone derivatives, N-isoamylsclerotiorinamine (**1**) and 7-methoxyl-*N*-isoamylsclerotiorinamine (**2**), and four known azaphilones (**3**–**6**), together with two new chromone derivatives, penithochromones X and Y (**7** and **8**). Their structures were elucidated based on spectroscopic data, CD spectrum, and semi-synthesis. Sclerotioramine (**3**) showed significant antibacterial activities against *B. subtilis* and *S. dysentery*, and it also showed most potent anti-plant pathogenic fungi activities against *P. theae*, *C. miyabeanus*, and *E. turcicum*.

## 1. Introduction

Azaphilones, known as fungal pigments, are a family of fungal polyketide metabolites with a highly oxygenated pyranoquinone bicyclic core [1,2,3]. They are mainly obtained from fungal genera including *Aspergillus* sp., *Talaromyces* sp., and *Penicillium* sp. [4,5,6]. These compounds have been proven with a variety of biological activities, such as antiviral activity, cytotoxicity, anti-inflammatory activity, as well as antimicrobial activity [7,8,9,10,11]. For example, chaephilone C and chaetoviridides A–C, which were isolated from a marine-derived fungus *Chaetomium* sp. NA-S01-R1, exhibited notable antibacterial activities against *Vibrio rotiferianus* and *V. vulnificus*, and anti-methicillin-resistant *Staphylococcus aureus* (anti-MRSA) activities [12]. Due to their structural diversity and promising bioactivity, azaphilones have received increased attention, and more than 600 naturally-derived azaphilones have been reported [13]. 

**Figure 1:**
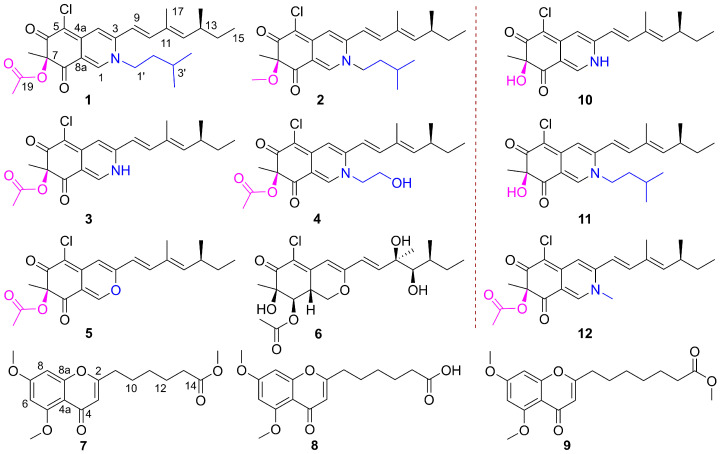
Structures of natural compounds **1**–**9**, and semi-synthetic compounds **10**–**12**.

Fungi are a promising source of novel and biologically active natural products for drug discovery [14]. *Penicillium* fungi, recognized for their ability to generate structurally novel and bioactive compounds [15,16], are an important source of antimicrobial agents. In our ongoing search for bioactive metabolites from endophytic fungi [17,18,19], a *Sophora tonkinensis*-associated fungus, *Penicillium* sp. GDGJ-N37, was investigated. The EtOAc extract of this fungus showed antibacterial activity to *Bacillus subtilis* and antifungal activity to *Setosphaeria turcica*. A follow-up chemical investigation of the extract led to the isolation of two new azaphilones, *N*-isoamylsclerotiorinamine (**1**) and 7-methoxyl-*N*-isoamylsclerotiorinamine (**2**), together with four known azaphilone derivatives, sclerotioramine (**3**) [20], isochromophilone VI (**4**) [21], sclerotiorin (**5**) [21], and hypocrellone A (**6**) [22]. Two new chromone derivatives, penithochromones X and Y (**7** and **8**), together with a known one, penithochromone F (**9**) (Figure 1) [23], were also obtained from the fungus. Among these azaphilones, **3** could be obtained by semi-synthesis from **5** in a yield over 30% by a one-step process. Additionally, azaphilone derivatives **10**–**12** were semi-synthesized for structure elucidation and structure–activity relationship (SAR) studies. Herein, we described the isolation, structure elucidation, and antimicrobial activity of these compounds. Preliminary SAR of the azaphilone derivatives were also discussed. 

## 2. Results and Discussion

*N*-Isoamylsclerotiorinamine (**1**) was obtained as a red amorphous powder. Its molecular formula was assigned as C_26_H_34_ClNO_4_ with 10 degrees of unsaturation based on the HRESIMS at *m*/*z* 460.2275 [M + H]^+^ (calcd. for C_26_H_35_ClNO_4_^+^, 460.2255). The intensity of an isotope peak at *m*/*z* 462.2255 (calcd. for C_26_H_35_^37^ClNO_4_^+^, 460.2220) is about 30%, indicating the presence of a chlorine atom in **1**. The ^1^H NMR spectrum of **1** (Table 1) presented seven methyls at *δ*_H_ 2.12 (3H, s), 1.92 (3H, d, *J* = 1.2 Hz), 1.50 (3H, s), 1.04 (3H, d, *J* = 6.4 Hz), 0.99 (6H, d, *J* = 6.0 Hz), and 0.90 (3H, t, *J* = 7.6 Hz), five olefinic protons at *δ*_H_ 8.18 (1H, s), 7.16 (1H, s), 7.12 (1H, d, *J* = 15.6 Hz), 6.46 (1H, d, *J* = 15.6 Hz), and 5.80 (1H, d, *J* = 9.6 Hz). The ^13^C NMR spectrum (Table 1) revealed the presence of 20 carbons, including two ketones (*δ*_C_ 194.9 and 185.4) and one ester carbonyl group (*δ*_C_ 171.6), ten olefinic carbons (*δ*_C_ 151.2, 149.0, 148.2, 146.5, 143.7, 133.7, 116.8, 116.7, 112.6, and 101.1), one oxygenated quaternary carbon (*δ*_C_ 86.2), two methine carbons (*δ*_C_ 36.2 and 27.3), three methylene carbons (*δ*_C_ 54.3, 40.0, and 31.2), and seven methyl carbons (*δ*_C_ 23.8, 22.7 × 2, 20.6, 20.2, 12.8, and 12.4). The combination of *δ*_H_ 0.99 (6H, d, *J* = 6.0 Hz) and *δ*_C_ 22.7 × 2 indicated the presence of an isopropyl group in the structure.

**Figure 2:**
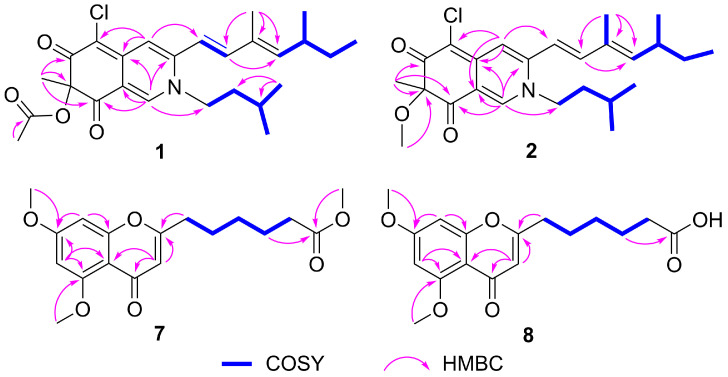
Key HMBC and COSY correlations of compounds **1**, **2**, **7**, and **8**.

The HMBC correlations (Figure 2) from H-1 to C-3, C-4a, C-8, and C-8a, from H-4 to C-4a and C-5, and from H-18 to C-6, C-7, and C-8, indicated the existence of an isoquinoline-6,8(2*H*,7*H*)-dione moiety, a typical structural core in azaphilone skeleton. The HMBC correlations (Figure 2) from H-10 to C-12, from H-17 to C-10, C-11, and C-12, combined with the ^1^H-^1^H COSY correlations between H-9/H-10, H-12/H-13/H-14/H-15, and H-13/H-16 indicated the presence of a 3,5-dimethyl-1,3-heptadiene group. This side chain moiety linked to C-3 was proved by the HMBC correlations from H-9 to C-3 and H-4 to C-9. Comparing the NMR data of **1** and sclerotioramine (**3**) [20] indicated that **1** was a sclerotioramine derivative. The major difference was that **1** had an isoamyl fragment located at N-2, which was confirmed by the HMBC correlations from H-1 to C-1’, from H-4’ /H-5’ to C-2’ and C-3’, and by the ^1^H-^1^H COSY correlations between H-1’/H-2’/H-3’/H-4’ and H-5’. Thus, the planar structure of **1** was established (Figure 1).

7-Methoxyl-*N*-isoamylsclerotiorinamine (**2**) was isolated as a red powder. The molecular formula was determined as C_25_H_34_ClNO_3_ with 9 degrees of unsaturation by the HRESIMS at *m*/*z* 432.2322 [M + H]^+^ (calcd. for C_25_H_35_ClNO_3_^+^, 432.2305). An isotope peak at *m*/*z* 434.2298 (calcd. for C_25_H_35_^37^ClNO_3_^+^, 434.2271) indicated the presence of a chlorine atom in **2**. The ^1^H NMR and ^13^C NMR data of **2** (Table 1) were similar to those of **1**, except that the acetyl group (*δ*_C_ 171.6, 20.2; *δ*_H_ 2.12) in **1** was replaced by a methoxy group (*δ*_C_ 54.9, *δ*_H_ 3.15) in **2**. This observation was further confirmed by the HMBC correlation (Figure 2) from H-19 (*δ*_H_ 3.15) to C-7 (*δ*_C_ 90.4). On the basis of the spectroscopic data, the planar structure of **2** was assigned as shown in Figure 1.

**Figure 3:**
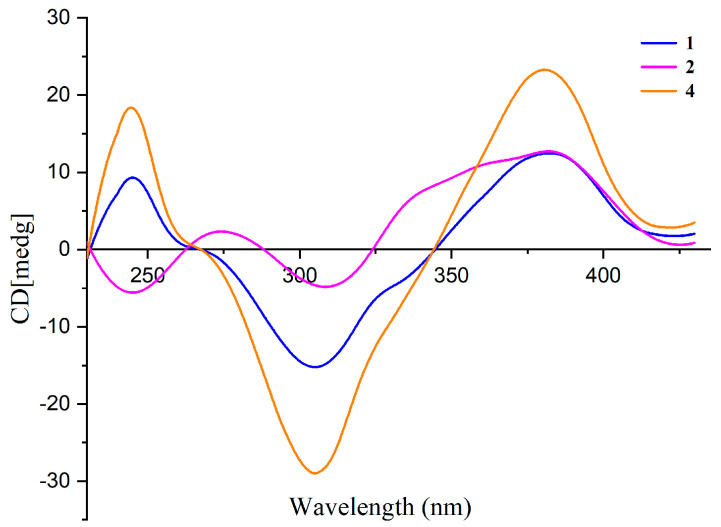
CD spectra of compounds **1**, **2**, and **4** in MeOH.

The absolute configuration at C-7 of **1** and **2** was elucidated by comparing their CD spectra with that of isochromophilone VI (**4**) (Figure 3). Compounds **1**, **2**, and **4** had similar CD spectra, which showed a positive Cotton effect at 380 nm and a negative Cotton effect at 300 nm. It revealed that the absolute configuration of C-7 was *R* in **1** and **2** [24,25]. The absolute configuration of the C-13 stereocenter in **1** and **2** was determined by semi-synthesis. Isoamylamine was employed to provide **1** and the deacetylate analogue **11** from the known sclerotiorin (**5**). Compound **11** was further methylated with CH_3_I to give **2**. The ^1^H NMR spectra of the semisynthetic products **1** and **2** were identical to those of the natural products **1** and **2**, respectively. On the other hand, compounds **1** and **2** are most likely derived from the same biogenetic pathway as **3** and **4**. It meant that the absolute configuration of C-13 in **1** and **2** was an *S*-configuration, just the same as the absolute configuration of C-13 in **3** and **4**. Thus, the absolute configurations of **1** and **2** were 7*R*, 13*S*.

Penithochromone X (**7**) was isolated as a light-yellow oil. Its molecular formula was established as C_18_H_22_O_6_ by HRESIMS at *m*/*z* 335.1490 [M + H]^+^ (calcd. for C_18_H_23_O_6_^+^, 335.1489), indicating 8 degrees of unsaturation. The ^1^H NMR spectrum of **10** (Table 2) revealed one pair of meta coupled protons [*δ*_H_ 6.40 (1H, d, *J* = 2.3 Hz) and 6.32 (1H, d, *J* = 2.3 Hz)], one olefinic proton [*δ*_H_ 5.98 (1H, s)], three methoxy groups [*δ*_H_ 3.91 (3H, s), 3.86 (3H, s), and 3.65 (3H, s)], and five methylenes [*δ*_H_ 2.50 (2H, t, *J* = 7.4 Hz), 2.31 (2H, t, *J* = 7.4 Hz), 1.72 (2H, m), 1.68 (2H, m), and 1.40 (2H, m)]. The ^13^C NMR spectrum (Table 2) displayed 18 carbon signals, including two carbonyl carbons (*δ*_C_ 177.7 and 174.1), eight olefinic carbons (*δ*_C_ 166.2, 163.9, 161.0, 160.3, 111.3, 109.1, 96.0, and 92.8), three methoxy carbons (*δ*_C_ 56.5, 55.8, and 51.6), and five methylene carbons (*δ*_C_ 33.9, 33.4, 28.5, 26.3, and 24.6). The HMBC correlations from H-3 to C-2, C-4, and C-4a, from H-6 to C-4a, C-5, and C-7, from H-8 to C-7 and C-8a, from 5-OCH_3_ to C-5, and 7-OCH_3_ to C-7, together with the remaining 7 degrees of unsaturation supported the existence of the 5,7-dioxygenated chromone moiety. The ^1^H-^1^H COSY correlations (Figure 2) of H-9/H-10/H-11/H-12/H-13, and the HMBC correlations (Figure 2) from H-12 to C-14, and from 14-OCH_3_ to C-14 defined the side chain. The HMBC correlation from H-9 to C-2 confirmed that the chain was located at C-2. The NMR data of **7** were similar to that of penithochromone F (**9**) [23], except for the disappearance of a CH_2_ unit in the side chain in **7**. Hence, the structure of **7** was assigned as shown in Figure 1.

Penithochromone Y (**8**) was isolated as a light-yellow oil. Its molecular formula was determined as C_17_H_20_O_6_ on the basis of HRESIMS analysis. Its NMR data resembled those of **7** (Table 2). The only distinction was the absence of 14-OCH_3_ in **8**. It was confirmed by the HMBC correlation from H-12 (*δ*_H_ 1.53) to C-14 (*δ*_C_ 174.7) (Figure 2). The structure of **8** is shown in Figure 1.

Semi-synthesis plays a pivotal role in providing enough material for further biological studies, determination of the absolute configurations, as well as investigation of the structure–activity relationship. During the study, the structure–activity relationship of these azaphilone derivatives was investigated. The semisynthetic transformation of **5** into **3** was achieved by one step using NH_3_·H_2_O [26], and deacelysclerotioramine (**10**) was also obtained as a byproduct. N-methylsclerotiorinamine (**12**) was semi-synthesized from **3** by methylating with CH_3_I [27].

The antibacterial activities of the natural products **1**–**9** and the semi-synthetic analogs **10**–**12** against *Staphylococcus aureus*, *Bacillus subtilis*, *Escherichia coli*, *Bacillus megaterium*, and *Shigella dysentery* were evaluated. As shown in Table S1, **3** showed antibacterial activities against *S. aureus*, *B. subtilis*, *B. megaterium,* and *S. dysentery* with MIC values of 12.5, 3.125, 3.125, and 6.25 μg/mL, respectively, while **5** was inactive to these five strains except for *B. subtilis* (MIC value 100 μg/mL). In light of the structures and antibacterial activity results, we could see that when the O-atom at the 2-position was replaced by a N-atom, just like compounds **5** and **3**, the bacterial activities would increase. It suggested that a N-atom at the 2-position in **3** was essential for its antibacterial activity. In addition, a comparison of the activities of **3** with **1**, **4** and **12** revealed that an alkyl group substitution of 2-NH might lose or decrease their antibacterial activities. It should be mentioned that all the tested compounds showed no inhibitory effect on *E. coli*.

The antifungal activities of all compounds except **9** against the five plant pathogenic fungi, *Alternaria citri*, *A. oleracea*, *Pestalotiopsis theae*, *Cochliobolus miyabeanus*, and *Exserohilum turcicum*, were tested. As shown in Table S2, **3** was found to exhibit significant antifungal activity against these fungi with MIC values ranging from 3.125 to 25 μg/mL surpassing the efficacy of the positive control carbendazim. Notably, **3** showed the most potent activity against *P. theae*, *C. miyabeanus*, and *E. turcicum*. Compound **5** exhibited **a** potent effect on *C. miyabeanus* and *E. turcicum* with MIC values of 6.25 and 12.5 μg/mL but showed inhibitory to *A. citri*, *A. oleracea*, and *P. theae* with MICs ranging from 50 to 100 μg/mL. These results indicated that a N-atom at the 2-position in **3** played a positive role in their antifungal activity. On the other hand, **3** displayed better activity than **1**, **4** and **12,** suggesting that the presence of 2-NH might increase its antifungal activity.

In the antimicrobial screening, sclerotioramine (**3**) exhibited significant antifungal efficacy, which is better than carbendazim. Compound **3** is a N-containing azaphilone, which was mainly obtained from *Penicillium* sp. and *Chaetomium* sp. [13]. It has demonstrated diverse biological activities, including anti-inflammatory [24], cytotoxic [28], and antibacterial activities [29]. However, few studies on the antifungal activity of **3** against plant pathogens have been reported. The present results contribute valuable insights into the potential applications of compound **3** as an effective antifungal agent.

## 3. Materials and Methods

### 3.1. General Experimental Procedures

The NMR spectral data were measured on Bruker AV 400 or 600 spectrometers (400 MHz for ^1^H NMR and 100 MHz for ^13^C NMR; 600 MHz for ^1^H NMR and 150 MHz for ^13^C NMR). Circular dichroism (CD) spectra were recorded on a JASCO J-1500 CD spectrometer (JASCO, Tokyo, Japan). Optical rotations were measured on a Bellingham-Stanley ADP 440+ polarimeter at 20 °C. The HRESI-MS data were measured on a Micro Mass Q-TOF spectrometer (Waters Corporation, Milford, MA, USA). Column chromatography was performed using silica gel (100–200 mesh, Qingdao Haiyang Chemical Co. Ltd., Qingdao, China), ODS (50 μm, YMC, Kyoto, Japan) and Sephadex LH-20 (GE) were used for column chromatography. Semi-preparative High Performance Liquid Chromatography (HPLC) was performed on a Shimadzu LC-20A system (Shimadzu Corporation, Tokyo, Japan) using an ODS column (250 × 10 mm, 5 μm, 2.0 mL/min, YMC).

### 3.2. Fungal Material

The fungus *Penicillium* sp. GDGJ-N37 was a *Sophora tonkinensis*-associated fungus obtained from Baise, Guangxi Province, China in 2017. The genomic DNA extraction was carried out using the Fungal DNA kits (E.Z.N.A., Omega, Norcross, GA, USA) in accordance with the manufacturer’s guidelines. The internal transcribed spacer (ITS1-5.8S-ITS2) regions of the fungi were amplified utilizing the polymerase chain reaction (PCR) with universal ITS primers, ITS1F (5′-CTTGGTCATTTAGAGGAAGTAA-3′) and ITS4 (5′-TCCTCCGCTTATTGATATGC-3′) [30]. The PCR involved an initial denaturation at 94 °C for 5 min, followed by 30 cycles of 94 °C denaturation for 40 s, 52 °C annealing for 40 s, and a 72 °C extension for 1 min, concluding with a final extension at 72 °C for 10 min. Subsequently, the amplified products underwent sequencing (Invitrogen, Shanghai, China), and a BLASTN search was employed to identify sequences with the closest match in the GenBank using the Basic Local Alignment Search Tool (NCBI). The sequence of its rDNA ITS region had been submitted to GenBank (the GenBank accession number OP622861). The strain was preserved at the State Key Laboratory for Chemistry and Molecular Engineering of Medicinal Resources, Guangxi Normal University.

### 3.3. Fermentation, Extraction and Isolation

The fungal strain was cultivated on rice solid medium in 270 Erlenmeyer flasks at room temperature for 30 days, each containing 80 g of rice and 100 mL of water. The fermented material was extracted with EtOAc (3 × 10 L) to afford the crude extract (90.0 g).

The extract was subjected to silica gel chromatography using a petroleum ether-ethyl acetate (100:0, 90:10, 70:30, 50:50, 30:70, 0:100) gradient system to give six fractions (Fr.1–Fr.6). Fr.1 (65.2 g) was isolated by silica gel chromatography using a petroleum ether-ethyl acetate (95:5, 90:10, 85:15, 80:20, 75:35, 70:30) gradient system to afford six subfractions (Fr.1.1–Fr.1.6). Fr.1.3 was purified by ODS eluting with MeOH-H_2_O (50:50–100:0) to yield **5** (12.0 g). Fr. 1.4 was chromatographed by ODS and semi-preparative HPLC (MeOH-H_2_O, 57:43) to afford **6** (40.6 mg). Fr.3 (12.5 g) was isolated by RP C18 with MeOH-H_2_O (40:60–100:0) gradient system to give four subfractions (Fr.3.1–Fr.3.4). Fr.3.3 (200.3 mg) was chromatographed on Sephadex LH-20 (MeOH) and semi-preparative HPLC (MeOH-H_2_O, 80:20) to afford **1** (8.2 mg) and **2** (4.1 mg). Fr.5 (9.3 g) was chromatographed by ODS and semi-preparative HPLC (MeOH-H_2_O, 76:24) to give **4** (10.2 mg). Fr.5 (500.6 mg) was isolated by ODS using a MeOH-H_2_O (45:55–100:0) to yield six fractions (Fr.5. 1–Fr.5. 6). Fr.5.6.4 (400.6 mg) was further purified by Sephadex LH-20 (CH_2_Cl_2_-CH_3_OH, 2:3) and semi-preparative HPLC (MeOH-H_2_O, 80:20) to afford **3** (100.0 mg). Fr.6 (3.3 g) was further purified by ODS using a MeOH-H_2_O (40:50–100:0) and to give five subfractions (Fr.6.1–Fr.6.5). Subfraction Fr.6.3 was further purified by Sephadex LH-20 (CH_2_Cl_2_-MeOH, 2:8) to afford subfractions Fr.6.3.1–Fr.6.3.4. Compound **9** (8.6 mg) was obtained from Fr.6.3.1 by semi-preparative HPLC (MeOH-H_2_O, 68:32). Fr.6.3.2 was purified by semi-preparative HPLC (MeOH-H_2_O, 58:42) to yield **8** (9.4 mg). Fr.6.3.4 was purified by semi-preparative HPLC (MeOH-H_2_O, 62:38) to give **7** (9.3 mg).

N-Isopenthysclerotiorinamine (**1**): red amorphous powder; ${\left[\alpha \right]}_{D}^{25}$ +196.6 (*c* 0.1, MeOH); UV (MeOH) λ_max_ (log *ε*) 230 (2.40), 362 (2.52); CD (0.4 mM, MeOH) λ_max_ (Δ*ε*) 246 (+3.7), 307 (−5.98), and 382 (+4.6) nm; ^1^H and ^13^C NMR data (Table 1); HRESIMS *m*/*z* 460.2256 [M + H]^+^ (calcd. for C_26_H_35_ClNO_4_^+^, 460.2255); 462.2249 (calcd. for C_26_H_35_^37^ClNO_4_^+^, 460.2220).

7-Methoxyl -N-isopenthysclerotiorinamine (**2**): red amorphous powder; ${\left[\alpha \right]}_{D}^{25}$ +188.4 (*c* 0.1, MeOH); UV (MeOH) λ_max_ (log *ε*) 230 (2.40), 362 (2.52); CD (0.4 mM, MeOH) λ_max_ (Δ*ε*) 247 (−3.5), 311 (−3.3), and 382 (+4.5) nm; ^1^H and ^13^C NMR data (Table 1); HRESIMS *m*/*z* 432.2314 [M + H]^+^ (calcd. for C_25_H_35_ClNO_3_^+^, 432.2305); 434.2294 (calcd. for C_25_H_35_^37^ClNO_3_^+^, 434.2271).

Penithochromone X (**7**): light yellow oil; UV (MeOH) λ_max_ (log *ε*) 246 (4.09), 292 (3.75) nm; ^1^H and ^13^C NMR data (Table 2); HRESIMS *m*/*z* 335.1514 [M + H]^+^ (calcd. for C_18_H_23_O_6_^+^, 335.1489); 373.1014 [M + K]^+^ (calcd. for C_18_H_22_O_6_K^+^, 373.1048).

Penithochromone Y (**8**): light yellow oil; UV (MeOH) λ_max_ (log *ε*) 250 (4.14), 290 (3.86) nm; ^1^H and ^13^C NMR data (Table 2); HRESIMS *m*/*z* 321.1341 [M + H]^+^ (calcd. for C_17_H_21_O_6_^+^, 321.1333); 343.1148 [M + Na]^+^ (calcd. for C_17_H_20_O_6_Na^+^, 343.1152).

### 3.4. General Procedure for the Semi-Synthesis of ***1***–***3***, and ***10***–***12***

Experimental details for **1** and **11**

A mixture of compound **5** (500.0 mg, 1.38 mmol, 1 equiv.) and excess isoamylamine (1.28 mL, 11.02 mmol, 8 equiv.) in reaction vials was stirred at 42 °C. The progress of the reaction was monitored by TLC. Upon completion, the reaction mixture was purified by silica gel column chromatography (EtOAc-petroleum, 25:75) and reverse-phase semi-preparative HPLC (MeOH-H_2_O, 20:80) to give **1** and its deacetylate analog **11**.

Compound **11:** amorphous powder; HRESIMS *m*/*z* 440.1982 [M + Na]^+^ (calcd. for C_24_H_32_ClNO_3_Na^+^, 440.1963); ^1^H NMR (400 MHz, CDCl_3_) *δ*_H_: 7.76 (1H, s, H-1), 7.02 (1H, d, *J* = 15.6 Hz, H-10), 7.01 (1H, s, H-4), 6.14 (1H, d, *J* = 15.6 Hz, H-9), 5.73 (1H, d, *J* = 9.6 Hz, H-12), 4.14 (1H, br s, 7-OH), 3.87 (2H, m, H-1’), 2.49 (1H, m, H-13), 1.85 (3H, d, *J* = 1.2 Hz, H-17), 1.69 (2H, m, H-2’), 1.66 (1H, m, H-3’), 1.55 (3H, s, H-18), 1.46 (1H, m, H-14α), 1.34 (1H, m, H-14β), 1.02 (3H, d, *J* = 6.6 Hz, H-16), 1.01 (3H, d, *J* = 5.6 Hz, H-4’), 0.99 (3H, d, *J* = 5.6 Hz, H-5’), 0.88 (3H, t, *J* = 7.4 Hz, H-15).

Experimental details for **2**

Compound **11** (50.0 mg, 119.6 µmol, 1 equiv.) and NaH (14.35 mg, 358.9 µmol, 3 equiv.) were dissolved in dry DMF (2 mL), CH_3_I (22.15 µL, 358.9 µmol, 3 equiv.) was then added, then the solution was stirred at 40 ℃ for 2 h. The reaction mixture was washed with an aqueous saturated NaHCO_3_ solution, and then the organic layer was evaporated to dryness to leave the crude product. The product was purified by silica gel column chromatography (EtOAc-petroleum, 25:75) and reverse-phase semi-preparative HPLC (MeOH-H_2_O, 20:80) to give **2**.

Experimental details for **3** and **10**

A mixture of compound **5** (500.0 mg, 1.28 mmol, 1 equiv.) and K_2_CO_3_ (571.3 mg, 4.13 mmol, 3 equiv.) in excess NH_3_·H_2_O (1.5 mL) was stirred at 50 ℃. The progress of the reaction was monitored by TLC. Upon completion, the reaction mixture was purified by silica gel column chromatography (EtOAc-petroleum, 75:25) and reverse-phase semi-preparative HPLC (MeOH-H_2_O, 80:20) to give **3** (129.3 mg) and its deacetylate analogue **10**.

Compound **10:** amorphous powder; HRESIMS *m*/*z* 348.1390 [M + H]^+^ (calcd. for C_19_H_23_ClNO_3_^+^, 348.1361); ^1^H NMR (400 MHz, CDCl_3_) *δ*_H_: 8.30 (1H, s, H-1), 7.27 (1H, d, *J* = 15.8 Hz, H-10), 6.98 (1H, s, H-4), 6.40 (1H, d, *J* = 15.8 Hz, H-9), 5.74 (1H, s, H-12), 2.47 (1H, br s, H-13), 1.87 (3H, s, H-17), 1.58 (3H, s, H-18), 1.41 (1H, s, H-14α), 1.30 (1H, m, H-14β), 0.99 (3H, d, *J* = 6.6 Hz, H-16), 0.86 (3H, t, *J* = 7.4, H-15).

Experimental details for **12**

Compound **12** was semi-synthesized from **3** by using a similar procedure as the conversion of compound **11** to **2**.

Compound **12:** amorphous powder; HRESIMS *m*/*z* 426.1462 [M + Na]^+^ (calcd. for C_22_H_26_ClNO_4_Na^+^, 426.1443); ^1^H NMR (400 MHz, CD_3_OD) *δ*_H_: 8.19 (1H, s, H-1), 7.22 (1H, s, H-4), 7.14 (1H, d, *J* = 15.5 Hz, H-10), 6.48 (1H, d, *J* = 15.5 Hz, H-9), 5.81 (1H, d, *J* = 9.8 Hz, H-12), 3.80 (3H, s, H-21), 2.55 (1H, m, H-13), 2.12 (3H, s, H-20), 1.94 (3H, s, H-17), 1.50 (3H, s, H-18), 1.46 (1H, m, H-14α), 1.36 (1H, m, H-14β), 1.04 (3H, d, *J* = 6.9 Hz, H-16), 0.90 (3H, t, *J* = 7.5 Hz, H-15). ^13^C NMR (100 MHz, CD_3_OD) *δ*_C_: 195.0 (C-8), 185.2 (C-6), 171.5 (C-19), 152.1 (C-12), 149.2 (C-5), 148.5 (C-10), 146.5 (C-3), 144.6 (C-1), 133.9 (C-11), 116.8 (C-8a), 116.4 (C-9), 112.0 (C-4), 100.9 (C-4a), 86.2 (C-7), 42.9 (C-1’), 36.3 (C-13), 31.2 (C-14), 23.8 (C-18), 20.6 (C-20), 20.2 (C-16), 12.7 (C-17), 12.4 (C-15).

### 3.5. Antimicrobial Assay

Antimicrobial evaluation against bacteria *Staphylococcus aureus*, *Bacillus subtilis*, *Escherichia coli*, *Bacillus megaterium*, and *Shigella dysentery* was carried out by the serial-dilution method following reports found in the literature [18,31,32]. Anti-phytopathogenic activities against *Alternaria citri*, *A. oleracea*, *Pestalotiopsis theae*, *Cochliobolus miyabeanus*, and *Exserohilum turcicum* were assessed using a modified version of the two-fold serial dilutions method as the literature described [33,34]. The test compounds were dissolved in DMSO to prepare a stock solution. Ciprofloxacin and carbendazim were used as the positive controls with respect to bacteria and plant pathogenic fungi.

## 4. Conclusions

In summary, we described a chemical investigation of the fungus *Penicillium* sp. GDGJ-N37. Two new nitrogenated azaphilones, N-isoamylsclerotiorinamine (**1**) and 7-methoxyl-N-isoamylsclerotiorinamine (**2**), together with four known azaphilones (**3**–**6**), and two new chromone derivatives, penithochromones X and Y (**7** and **8**), were obtained from the fermentation culture of the fungus. Remarkably, compound **3** exhibited significant anti-plant pathogenic fungi activities. The present research not only expands the structural diversity of azaphilones, but also provides inspiration for the discovery of antifungal leading compounds.

## Figures and Tables

**Table 1:** ^1^H and ^13^C NMR assignments for compounds **1** and **2** (CD_3_OD).

No.		1 *^a^*		2 *^b^*
	*δ*_C_	*δ*_H_ (*J* in Hz)	*δ*_C_	*δ*_H_ (*J* in Hz)
1	143.7	8.18, s	143.2	8.19, s
3	148.2		148.9	
4	112.6	7.16, s	112.5	7.18, s
4a	151.2		151.7	
5	101.1		102.2	
6	185.4		186.5	
7	86.2		90.4	
8	194.9		199.0	
8a	116.7		117.7	
9	116.8	6.46, d (15.6)	116.7	6.48, d (15.6)
10	146.5	7.12, d (15.6)	146.7	7.14, d (15.6)
11	133.7		133.7	
12	149.0	5.80, d (9.6)	149.2	5.81, d (9.6)
13	36.2	2.55, m	36.3	2.55, m
14	31.2	1.46, m	31.2	1.49, m
		1.36, m		1.36, m
15	12.4	0.90, t (7.6)	12.4	0.90, t (7.2)
16	20.6	1.04, d (6.4)	20.6	1.04, d (6.8)
17	12.8	1.92, d (1.2)	12.7	1.93, d (1.2)
18	23.8	1.50, s	27.5	1.46, s
19	171.6		54.9	3.15, s
20	20.2	2.12, s		
1’	54.3	4.14, t (8.4)	54.4	4.17, t (8.0)
2’	40.0	1.67, overlapped	40.0	1.69, overlapped
3’	27.3	1.67, overlapped	27.1	1.69, overlapped
4’/5’	22.7	0.99, d (6.0)	22.7	1.01, d (5.6)

*^a^* ^1^H NMR measured at 400 MHz; ^13^C NMR measured at 100 MHz. *^b^* ^1^H NMR measured at 600 MHz; ^13^C NMR measured at 150 MHz.

**Table 2:** ^1^H and ^13^C NMR assignments for compounds **7** (CDCl_3_) and **8** (DMSO-*d*_6_).

No.	7		8	
	*δ*_C_	*δ*_H_ (*J* in Hz)	*δ*_C_	*δ*_H_ (*J* in Hz)
2	166.2		166.1	
3	111.3	5.98, s	110.6	5.93, s
4	177.7		175.5	
4a	109.1		108.1	
5	161.0		160.3	
6	96.0	6.32, d (2.3)	96.1	6.46, d (2.3)
7	163.9		163.5	
8	92.8	6.40, d (2.3)	93.0	6.62, d (2.3)
8a	160.3		160.0	
9	33.4	2.50, t (7.4)	32.4	2.52, t (7.4)
10	26.3	1.72, m	25.8	1.63, m
11	28.5	1.40, m	27.9	1.33, m
12	24.6	1.68, m	24.2	1.53, m
13	33.9	2.31, t (7.4)	33.7	2.20, t (7.4)
14	174.1		174.7	
5-OCH_3_	56.5	3.91, s	56.1	3.79, s
7-OCH_3_	55.8	3.86, s	55.9	3.85, s
14-OCH_3_	51.6	3.65, s		

^1^H NMR measured at 400 MHz; ^13^C NMR measured at 100 MHz.

## Data Availability

The data presented in this study are available in the Supplementary Materials or can be provided by the authors.

## Supplementary Materials

The following supporting information can be downloaded at: https://www.mdpi.com/article/10.3390/molecules29020348/s1, Spectroscopic data of compounds **3**–**6**; Table S1. Antibacterial activities of compounds **1**–**12** (MIC, µg/mL); Table S2. Antifungal activities of compounds **1**–**8**, and **10**–**12** (MIC, µg/mL); Figure S1. HR-ESI-MS spectrum of compound **1**; Figure S2. ^1^H NMR (400 MHz, CD_3_OD) spectrum of compound **1**; Figure S3. ^13^C NMR (100 MHz, CD_3_OD) spectrum of compound **1**; Figure S4. HMQC (CD_3_OD) spectrum of compound **1**; Figure S5. ^1^H-^1^H COSY (CD_3_OD) spectrum of compound **1**; Figure S6. HMBC (CD_3_OD) spectrum of compound **1**; Figure S7. HR-ESI-MS spectrum of compound **2**; Figure S8. ^1^H NMR (600 MHz, CD_3_OD) spectrum of compound **2**; Figure edS9. ^13^C NMR (150 MHz, CD_3_OD) spectrum of compound **2**; Figure S10. HMQC (CD_3_OD) spectrum of compound **2**; Figure S11. ^1^H-^1^H COSY (CD_3_OD) spectrum of compound **2**; Figure S12. HMBC (CD_3_OD) spectrum of compound **2**; Figure S13. HR-ESI-MS spectrum of compound **7**; Figure S14. ^1^H NMR (400 MHz, CDCl_3_) spectrum of compound **7**; Figure S15. ^13^C NMR (100 MHz, CDCl_3_) spectrum of compound **7**; Figure S16. HMQC (CDCl_3_) spectrum of compound **7**; Figure S17. ^1^H-^1^H COSY (CDCl_3_) spectrum of compound **7**; Figure S18. HMBC (CDCl_3_) spectrum of compound **7**; Figure S19. HR-ESI-MS spectrum of compound **8**; Figure S20. ^1^H NMR (400 MHz, DMSO-*d*_6_) spectrum of compound **8**; Figure S21. ^13^C NMR (100 MHz, DMSO-*d*_6_) spectrum of compound **8**; Figure S22. HMQC (DMSO-*d*_6_) spectrum of compound **8**; Figure S23. ^1^H-^1^H COSY (DMSO-*d*_6_) spectrum of compound **8**; Figure S24. HMBC (DMSO-*d*_6_) spectrum of compound **8**.
