# Supplementary material for: Isolation and Characterization of Antimicrobial Metabolites from the Sophora tonkinensis-Associated Fungus Penicillium sp. GDGJ-N37
Source: Molecules. 2024 Jan 10;29(2):348. doi: 10.3390/molecules29020348 (PMC10818566; doi:10.3390/molecules29020348)
Supplement: Supplementary file 1 [file molecules-29-00348-s001.zip › molecules-2777959-supplementary.pdf]

Supplementary materials

Isolation and Characterization of Antimicrobial Metabolites from the *Sophora tonkinensis***-Associated Fungus** ***Penicillium*** **sp. GDGJ-N37**

Lili Huang, Yongxia Li, Jing Pang, Liuxia Lv, Jiatong Zhou, Liqi Liang, Xianhua He, Jun Li, Weifeng Xu * and Ruiyun Yang *

State Key Laboratory for Chemistry and Molecular Engineering of Medicinal Resources, School of Chemistry and Pharmaceutical Sciences, Guangxi Normal University, Guilin 541004, China

Corresponding Author

*E-mail: yxuweifeng_u@mailbox.gxnu.edu.cn (W.X.);

yang_rui_yun@mailbox.gxnu.edu.cn (R.Y.)

ABSTRACT: Chemical investigation of *Penicillium* sp. GDGJ-N37, a *Sophora tonkinensis*-associated fungus, yielded two new azaphilone derivatives, N-isoamylsclerotiorinamine (**1**) and 7-methoxyl-*N*-isoamylsclerotiorinamine (**2**), and four known azaphilones (**3**–**6**), together with two new chromone derivatives, penithochromones X and Y (**7** and **8**). Their structures were elucidated based on spectroscopic data, CD spectrum, and semi-synthesis. Sclerotioramine (**3**) showed significant antibacterial activities against *B. subtilis* and *S. dysentery*, and it also showed most potent anti-plant pathogenic fungi activities against *P. theae*, *C. miyabeanus*, and *E. turcicum*.

KEYWORDS: *Sophora tonkinensis; Penicillium* sp.; azaphilone derivatives; antibacterial activities; antifungal activities.

List of supporting information

Spectroscopic data of compounds **3–6**

Table S1. Antibacterial activities of compounds **1–12** (MIC, μg/mL)

Table S2. Antifungal activities of compounds **1–8**, and **10–12** (MIC, *μ*g/mL)

Figure S1. HR-ESI-MS spectrum of compound **1**

Figure S2. ^{1}H NMR (400 MHz, CD_3OD) spectrum of compound **1**

Figure S3. ^{13}C NMR (100 MHz, CD_3OD) spectrum of compound **1**

Figure S4. HMQC (CD_3OD) spectrum of compound **1**

Figure S5. ^{1}H-^{1}H COSY (CD_3OD) spectrum of compound **1**

Figure S6. HMBC (CD_3OD) spectrum of compound **1**

Figure S7. HR-ESI-MS spectrum of compound **2**

Figure S8. ^{1}H NMR (600 MHz, CD_3OD) spectrum of compound **2**

Figure S9. ^{13}C NMR (150 MHz, CD_3OD) spectrum of compound **2**

Figure S10. HMQC (CD_3OD) spectrum of compound **2**

Figure S11. ^{1}H-^{1}H COSY (CD_3OD) spectrum of compound **2**

Figure S12. HMBC (CD_3OD) spectrum of compound **2**

Figure S13. HR-ESI-MS spectrum of compound **7**

Figure S14. ^{1}H NMR (400 MHz, $CDCl_3$) spectrum of compound **7**

Figure S15. ^{13}C NMR (100 MHz, $CDCl_3$) spectrum of compound **7**

Figure S16. HMQC ($CDCl_3$) spectrum of compound **7**

Figure S17. ^{1}H-^{1}H COSY ($CDCl_3$) spectrum of compound **7**

Figure S18. HMBC ($CDCl_3$) spectrum of compound **7**

Figure S19. HR-ESI-MS spectrum of compound **8**

Figure S20. ^{1}H NMR (400 MHz, DMSO-d_6) spectrum of compound **8**

Figure S21. ^{13}C NMR (100 MHz, DMSO-d_6) spectrum of compound **8**

Figure S22. HMQC (DMSO-d_6) spectrum of compound **8**

Figure S23. ^{1}H-^{1}H COSY (DMSO-d_6) spectrum of compound **8**

Figure S24. HMBC (DMSO-d_6) spectrum of compound **8**

Spectroscopic data of compounds 3–6

Sclerotioramine (3): red amorphous powder; HRESIMS *m/z* 412.1290 [M + Na]$^+$ (calcd. for $C_{21}H_{24}ClNO_4Na^+$, 412.1286). ^{1}H NMR (400 MHz, CD_3OD) δ_H 8.03 (1H, s, H-1), 7.17 (1H, d, *J* = 16.2 Hz, H-10), 7.10 (1H, s, H-4), 6.24 (1H, d, *J* = 16.2 Hz, H-9), 5.72 (1H, d, *J* = 9.7 Hz, H-12), 2.55 (1H, m, H-13), 2.12 (3H, s, H-20), 1.91 (3H, d, *J* = 1.2 Hz, H-17), 1.51 (3H, s, H-18), 1.46 (1H, m, H-14a), 1.34 (1H, m, H-14b), 1.03 (3H, d, *J* = 6.6 Hz, H-16), 0.89 (1H, t, *J* = 7.4 Hz, H-15). ^{13}C NMR (100 MHz, CD_3OD) δ_C, 195.1 (C-8), 184.0 (C-6), 171.6 (C-19), 149.3 (C-4a), 148.9 (C-12), 148.0 (C-3), 143.3(C-10), 139.6 (C-1), 133.8 (C-11), 118.4 (C-9), 115.7 (C-8a), 112.4 (C-4), 101.9 (C-5), 86.1 (C-7), 36.2 (C-13), 31.2 (C-14), 23.9 (C-18), 20.6 (C-16), 20.2 (C-20), 12.5 (C-17), 12.3 (C-15).

Isochromophilone VI (4): red amorphous powder; HRESIMS *m/z* 434.1733 [M + H]$^+$ (calcd. for $C_{23}H_{29}ClNO_5Na^+$, 434.1734). ^{1}H NMR (400 MHz, CD_3OD) δ_H 8.16 (1H, s, H-1), 7.18 (1H, s, H-4), 7.10 (1H, d, *J* = 15.4 Hz, H-10), 6.56 (1H, d, *J* = 15.4 Hz, H-9), 5.78 (1H, d, *J* = 9.8 Hz, H-12), 4.24 (2H, dd, *J* = 5.2, 4.8 Hz, H-1'), 3.92 (2H, t, *J* = 5.0 Hz, H-2'), 2.55 (1H, m, H-13), 2.12 (3H, s, H-20),1.92 (3H, d, *J* = 1.2 Hz, H-17), 1.51 (3H, s, H-18), 1.46 (1H, m, H-14a), 1.34 (1H, m, H-14b), 1.04 (3H, d, *J* = 6.6 Hz, H-16), 0.90 (3H, t, *J* = 7.4, H-15). ^{13}C NMR (100 MHz, CD_3OD) δ_C,195.1 (C-8), 185.5 (C-6), 171.5 (C-19), 151.9 (C-5), 148.8 (C-12), 148.4 (C-3), 146.5 (C-10), 144.5 (C-1), 133.9 (C-11), 117.2 (C-9), 116.3 (C-8a), 112.6 (C-4), 101.2 (C-4a), 86.2 (C-7), 61.1 (C-2'), 57.5 (C-1'), 36.2 (C-13), 31.2 (C-14), 23.8 (C-18), 20.6 (C-16), 20.2 (C-20), 12.7 (C-17), 12.4 (C-15).

Sclerotiorin (5): yellow amorphous powder; ^{1}H NMR (400 MHz, CD_3OD) δ_H 8.19 (1H, s, H-1), 7.18 (1H, d, *J* = 15.7 Hz, H-10), 6.87 (1H, s, H-4), 6.37 (1H, d, *J* = 15.7 Hz, H-9), 5.74 (1H, d, *J* = 9.8 Hz, H-12), 2.54 (1H, m, H-13), 2.12 (3H, s, H-19), 1.89 (3H, d, *J* = 1.2 Hz, H-17), 1.54 (3H, s, H-18), 1.47 (1H, s, H-14a), 1.35 (1H, m, H-14b), 1.02 (3H, d, *J* = 6.6 Hz, H-16), 0.88 (3H, t, *J* = 7.3, H-15).

Hypocrellone A (6): yellow oil; HRESIMS *m/z* 429.1676 [M + H]$^+$ (calcd. for $C_{21}H_{30}ClNO_7^+$, 429.1675). ^{1}H NMR (400 MHz, CD_3OD) δ_H 6.75 (1H, d, *J* = 15.6 Hz, H-10), 6.27 (1H, d, *J* = 15.6 Hz, H-9), 6.11 (1H, s, H-4), 5.47 (1H, d, *J* = 2.9 Hz, H-8), 4.53 (1H, dd, *J* = 10.6, 4.9 Hz, H-1α), 3.79 (1H, dd, *J* = 13.0, 10.6 Hz, H-1β), 3.44 (1H, m, H-8a), 3.42 (1H, d, *J* = 2.2 Hz, H-12), 2.02 (3H, s, H-20), 1.72 (1H, m, H-13), 1.44 (1H, m, H-14α), 1.42 (3H, s, H-18), 1.31 (3H, s, H-17), 1.26 (1H, m, H-14β), 0.90 (3H, t, *J* = 7.4 Hz, H-15), 0.84 (3H, d, *J* = 6.8 Hz, H-16). ^{13}C NMR (100 MHz, CD_3OD) δ_C 193.7 (C-6), 172.3 (C-19), 163.2 (C-3), 145.8 (C-4a), 145.6 (C-10), 122.4 (C-9), 118.3 (C-5), 102.3 (C-4), 81.0 (C-12), 77.5 (C-11), 76.9 (C-8), 76.8 (C-7), 68.6 (C-1), 38.0 (C-8a), 36.1 (C-13), 30.0 (C-14), 26.5 (C-17), 24.1 (C-18), 20.6 (C-20), 14.3 (C-16), 12.2 (C-15).

Penithochromone F (9): light white solid; HRESIMS *m/z* 349.1631 [M + H]$^+$ (calcd. for $C_{19}H_{25}O_6^+$, 349.1646). ^{1}H NMR (400 MHz, acetone-*d*$_6$) δ_H 6.56 (1H, d, *J* = 2.3 Hz, H-8), 6.44 (1H, d, *J* = 2.3 Hz, H-6), 5.89 (1H, s, H-3), 3.91(3H, s, 7-OCH_3), 3.85 (3H, s, 5-OCH_3), 3.65 (3H, s, 14-OCH_3), 2.56 (2H, t, *J* = 7.4 Hz, H-9), 2.30 (2H, t, *J* = 7.4 Hz, H-14), 1.72 (2H, m, H-10), 1.61 (2H, m, H-13), 1.42 (2H, m, H-12), 1.40 (2H, m, H-11). ^{13}C NMR (100 MHz, acetone-*d*$_6$) δ_C 176.5 (C-4), 174.1 (C-14), 166.9 (C-2), 164.8 (C-7), 161.8 (C-5), 161.0 (C-8a), 111.7 (C-3), 109.6 (C-4a), 96.6 (C-6), 93.6 (C-8), 56.4 (5-OCH_3), 56.2 (14-OCH_3), 51.5 (7-OCH_3), 34.2 (C-9), 33.8 (C-14), 30.3 (C-11), 29.4 (C-12), 27.2 (C-10), 25.4 (C-13).

Table S1. Antibacterial activities of compounds **1–12** (MIC, μg/mL).

Compounds	*S. aureus*	*B. subtilis*	*E. coli*	*B. megaterium*	*S. dysentery*
**1**	I	I	I	I	I
**2**	I	I	I	I	I
**3**	12.5	3.125	I	3.125	6.25
**4**	25	50	I	25	50
**5**	I	100	I	I	I
**6**	100	I	I	50	I
**7**	I	I	I	I	I
**8**	I	I	I	I	I
**9**	I	I	I	I	I
**10**	I	I	I	I	I
**11**	50	50	I	–	I
**12**	I	I	I	I	I
Ciprofloxacin	25	0.78	6.25	6.25	6.25

I: Inactive at the concentration of 100 μg/mL.

Table S2. Antifungal activities of compounds **1–8**, and **10–12** (MIC, μg/mL).

Compounds	*A. citri*	*A. oleracea*	*P. theae*	*C. miyabeanus*	*E. turcicum*
**1**	I	I	I	I	I
**2**	I	I	I	I	I
**3**	12.5	25	6.25	6.25	3.125
**4**	25	I	25	50	100
**5**	50	100	50	6.25	12.5
**6**	50	50	I	I	I
**7**	I	I	I	I	I
**8**	I	I	I	I	I
**10**	I	I	I	I	I
**11**	I	I	I	I	I
**12**	50	25	50	I	I
Carbendazim	50	50	50	50	6.25

I: Inactive at the concentration of 100 μg/mL.

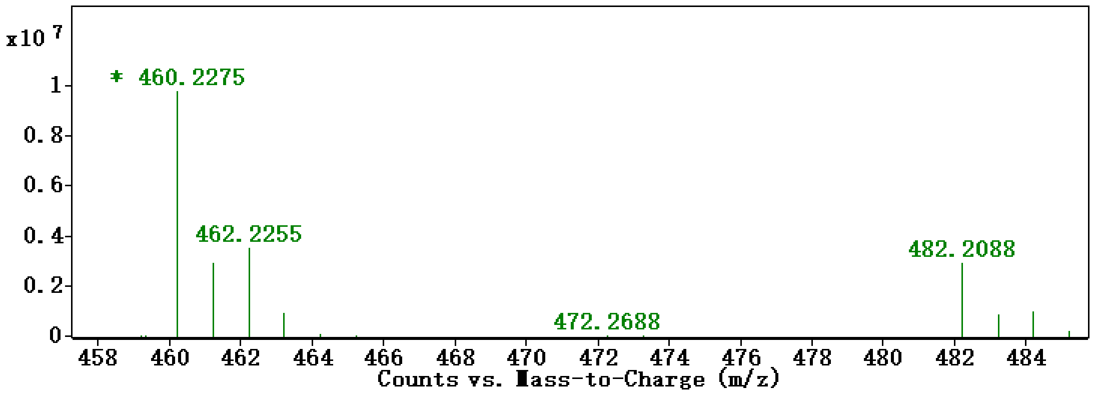

Figure S1. HR-ESI-MS spectrum of compound **1**

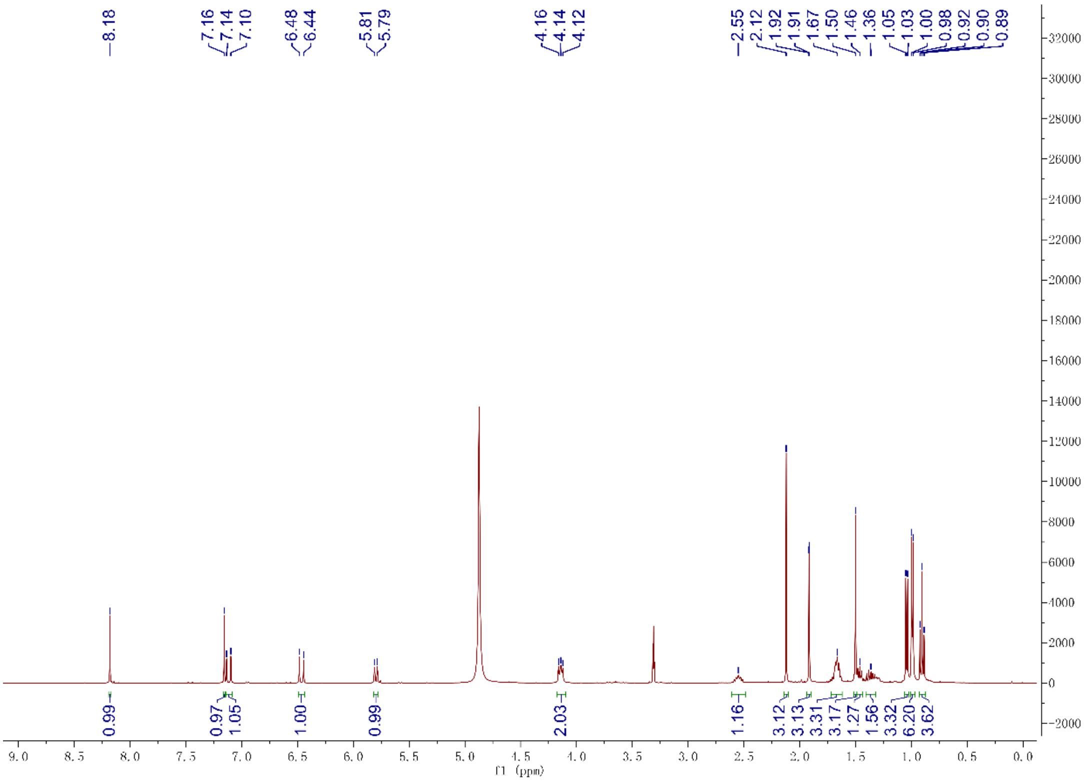

Figure S2. ^{1}H NMR (400 MHz, CD_3OD) spectrum of compound **1**

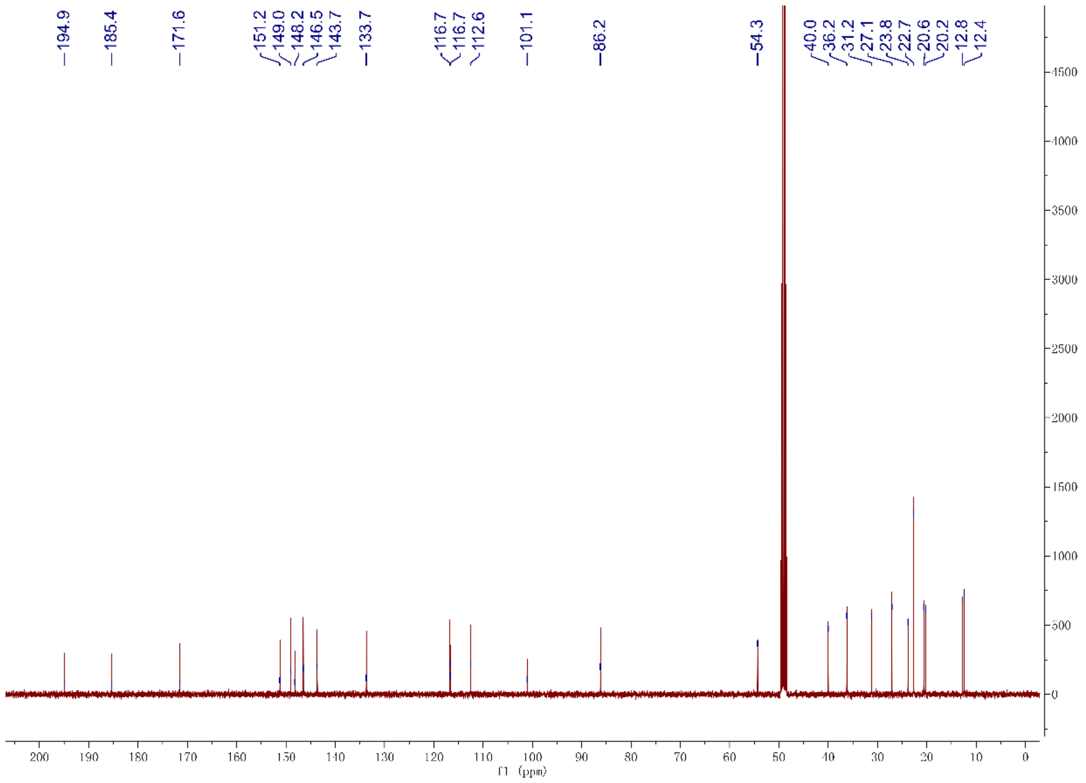

Figure S3. ^{13}C NMR (100 MHz, CD_3OD) spectrum of compound **1**

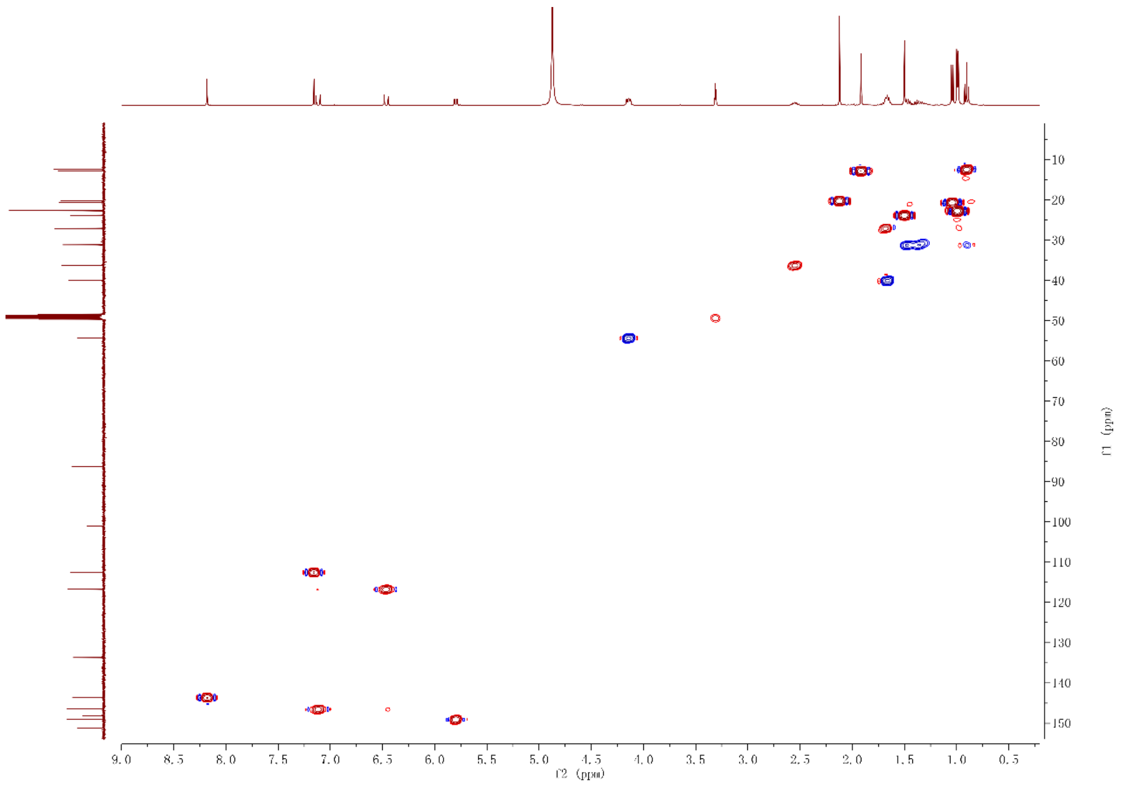

Figure S4. HMQC (CD_3OD) spectrum of compound **1**

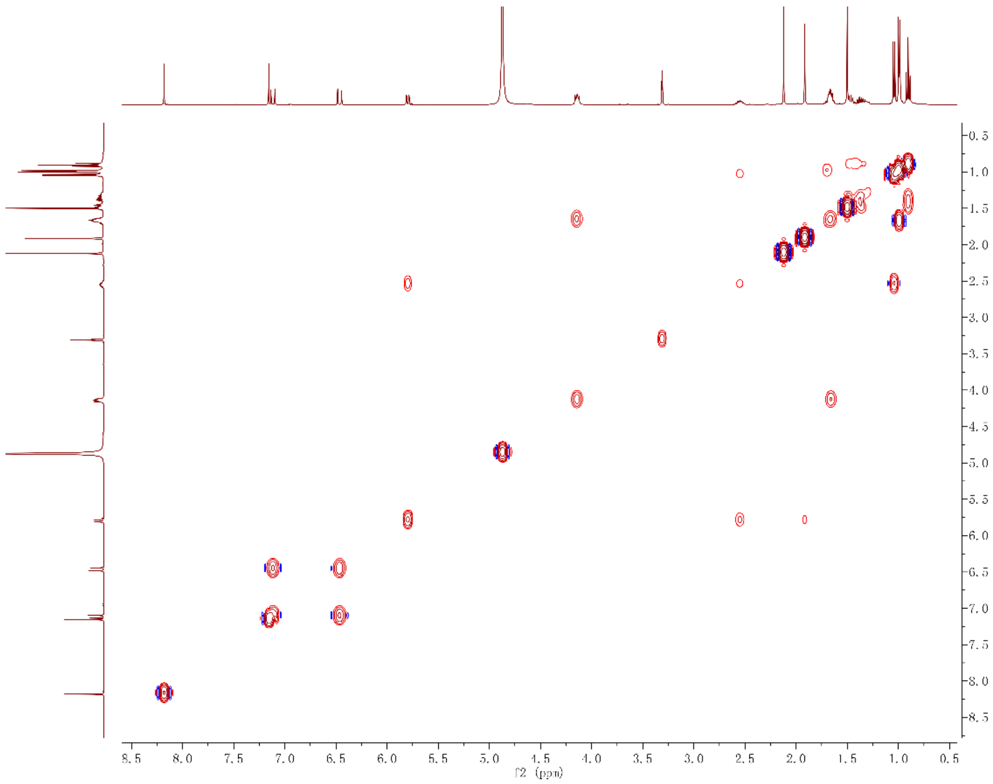

Figure S5. ^{1}H-^{1}H COSY (CD_3OD) spectrum of compound **1**

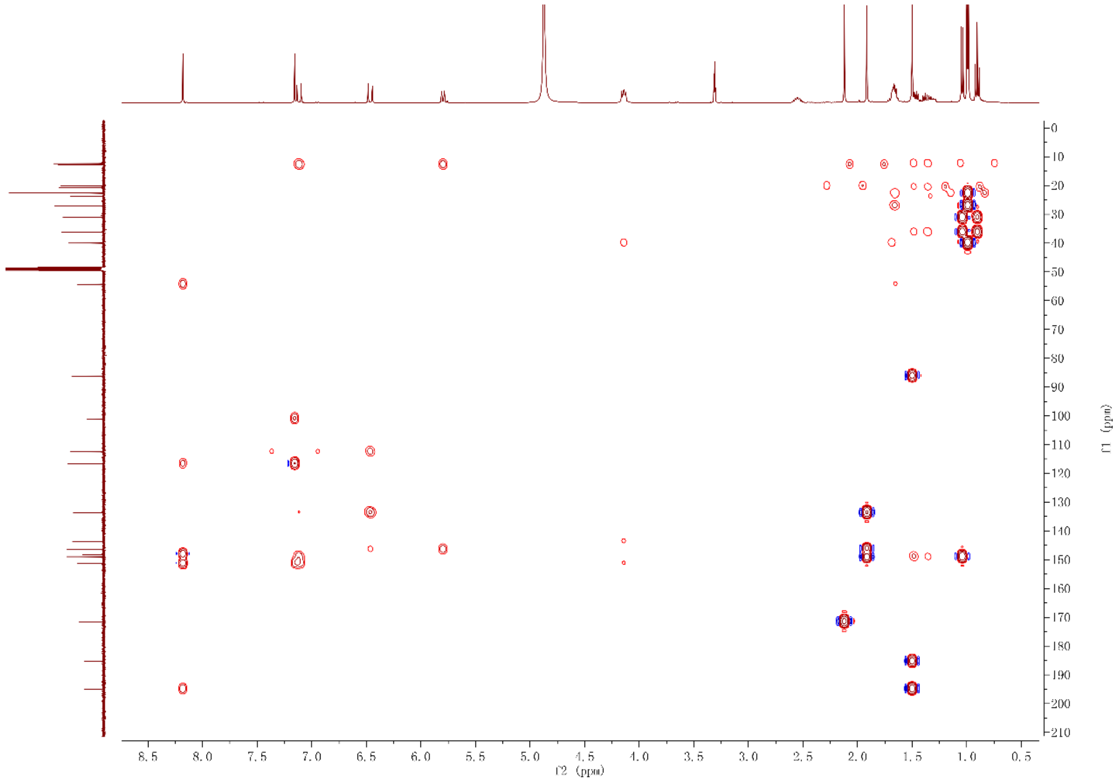

Figure S6. HMBC (CD_3OD) spectrum of compound **1**

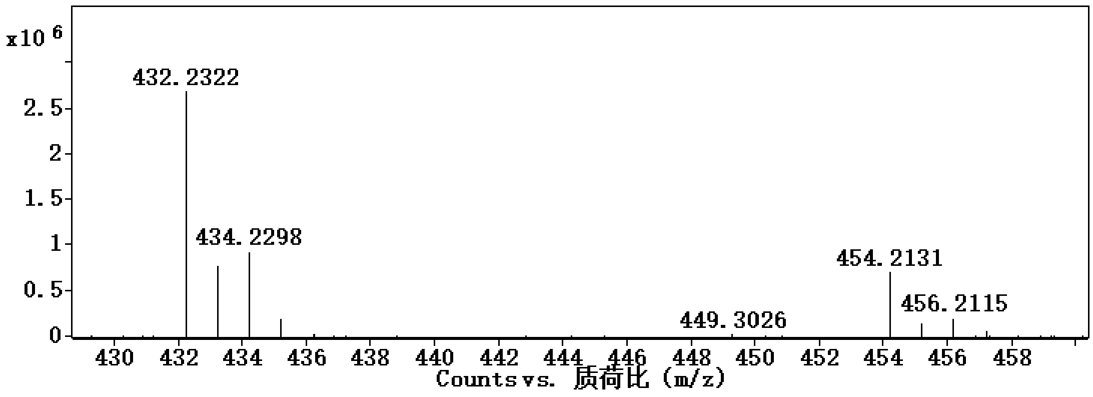

Figure S7. HR-ESI-MS spectrum of compound **2**

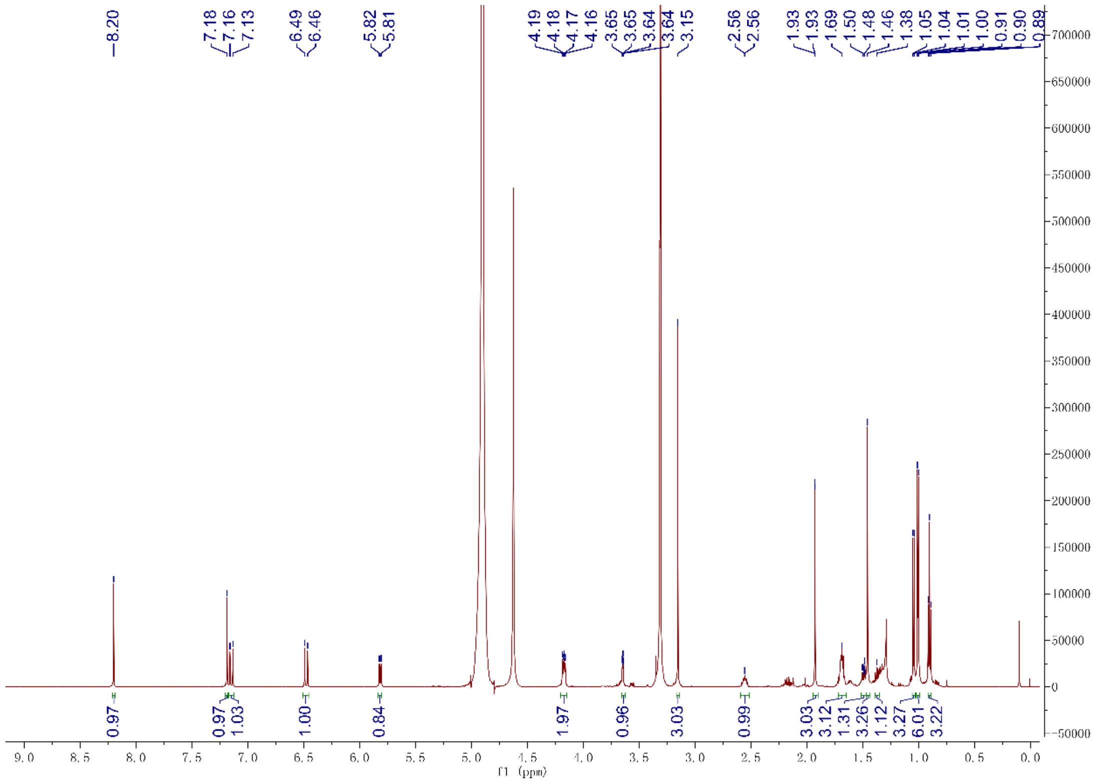

Figure S8. ^{1}H NMR (600 MHz, CD_3OD) spectrum of compound **2**

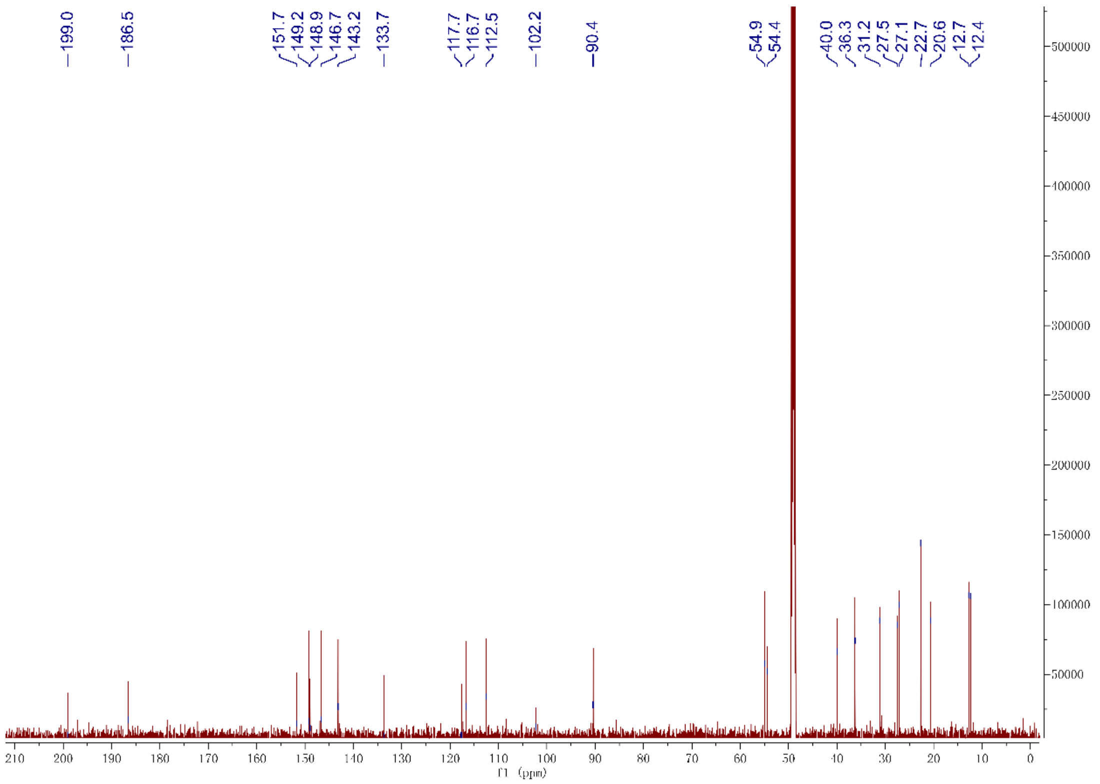

Figure S9. ^{13}C NMR (150 MHz, CD_3OD) spectrum of compound **2**

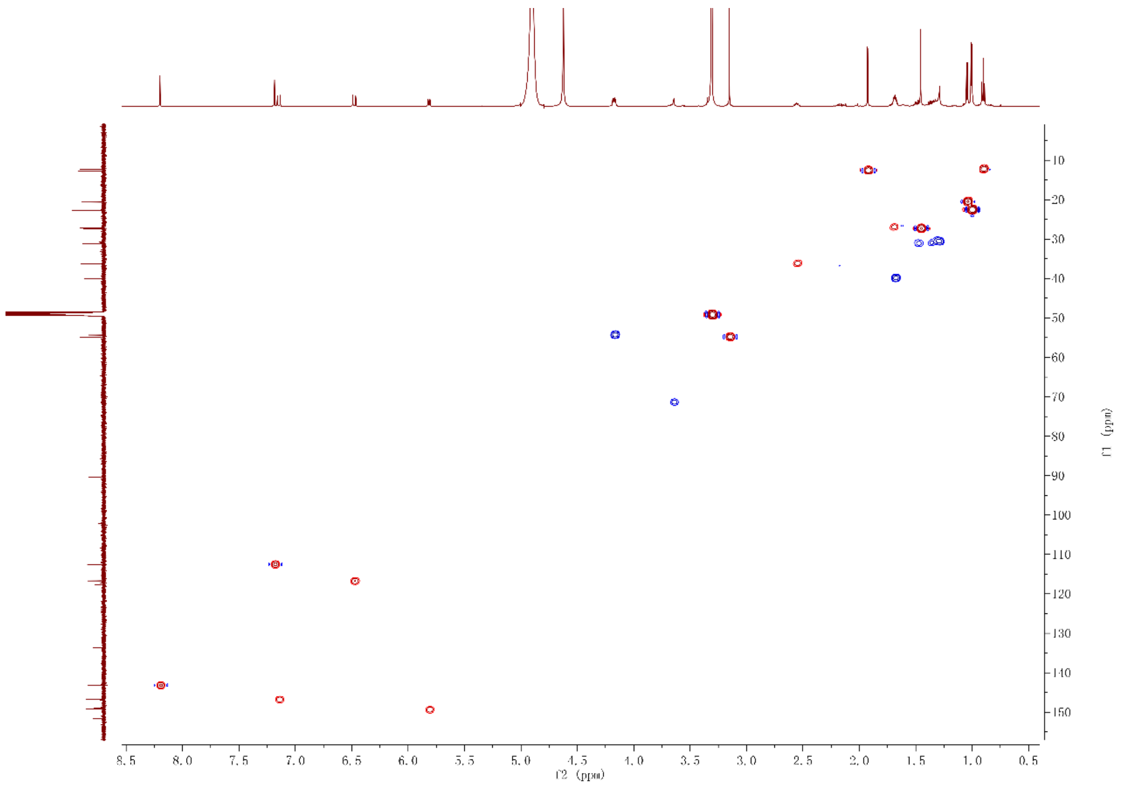

Figure S10. HMQC (CD_3OD) spectrum of compound **2**

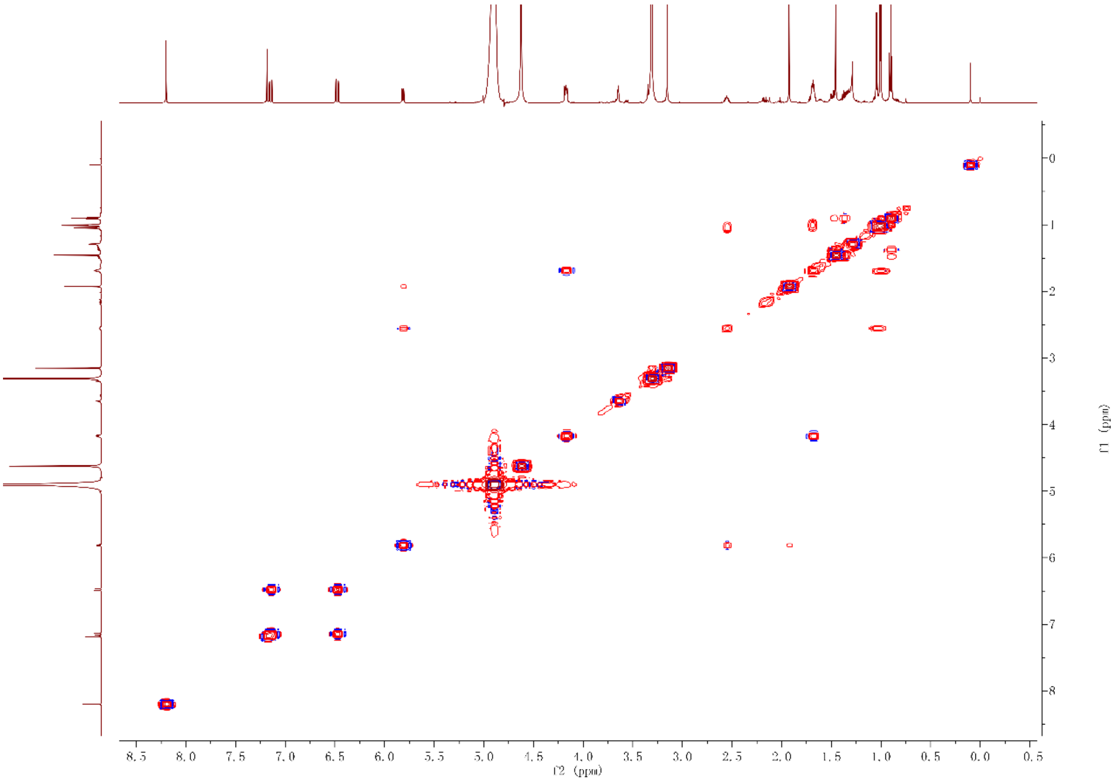

Figure S11. ^{1}H-^{1}H COSY (CD_3OD) spectrum of compound **2**

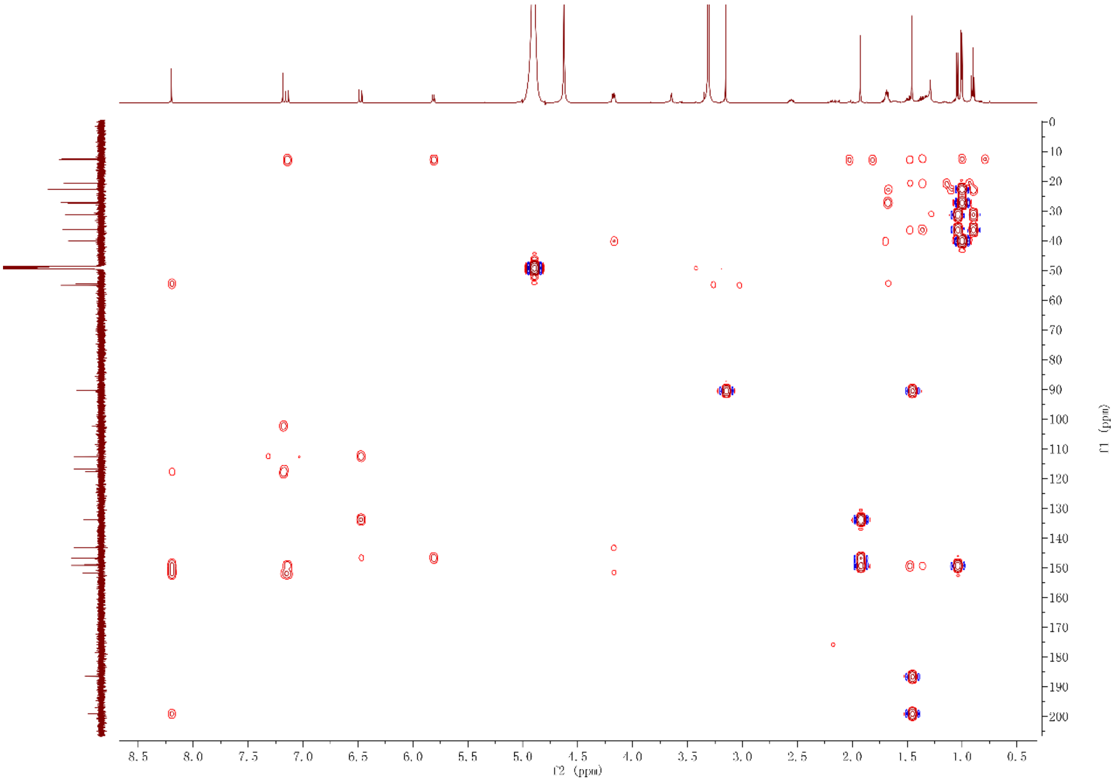

Figure S12. HMBC (CD_3OD) spectrum of compound **2**

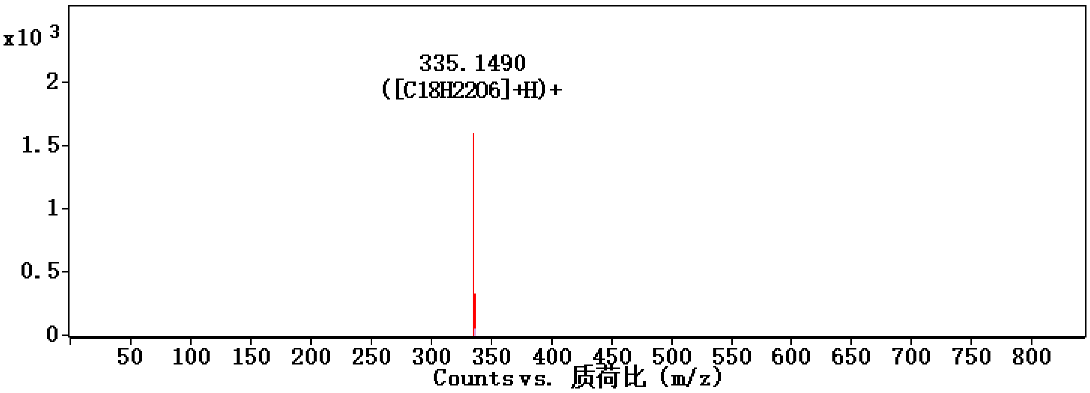

Figure S13. HR-ESI-MS spectrum of compound **7**

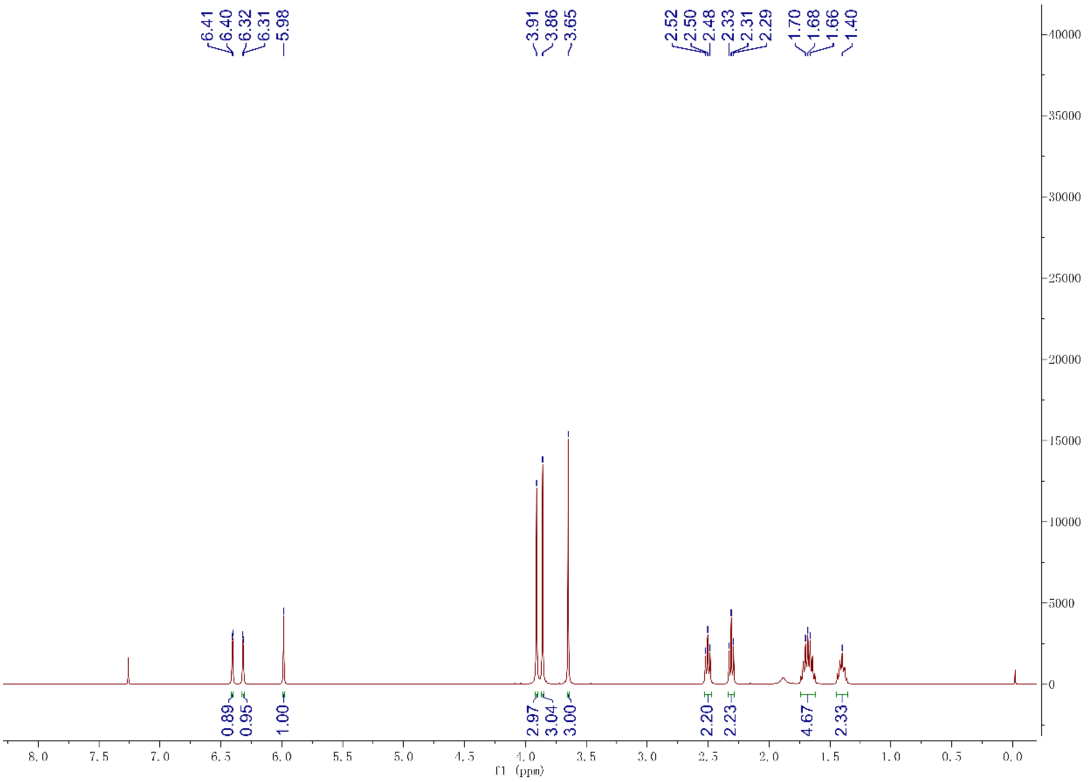

Figure S14. ^{1}H NMR (400 MHz, $CDCl_3$) spectrum of compound **7**

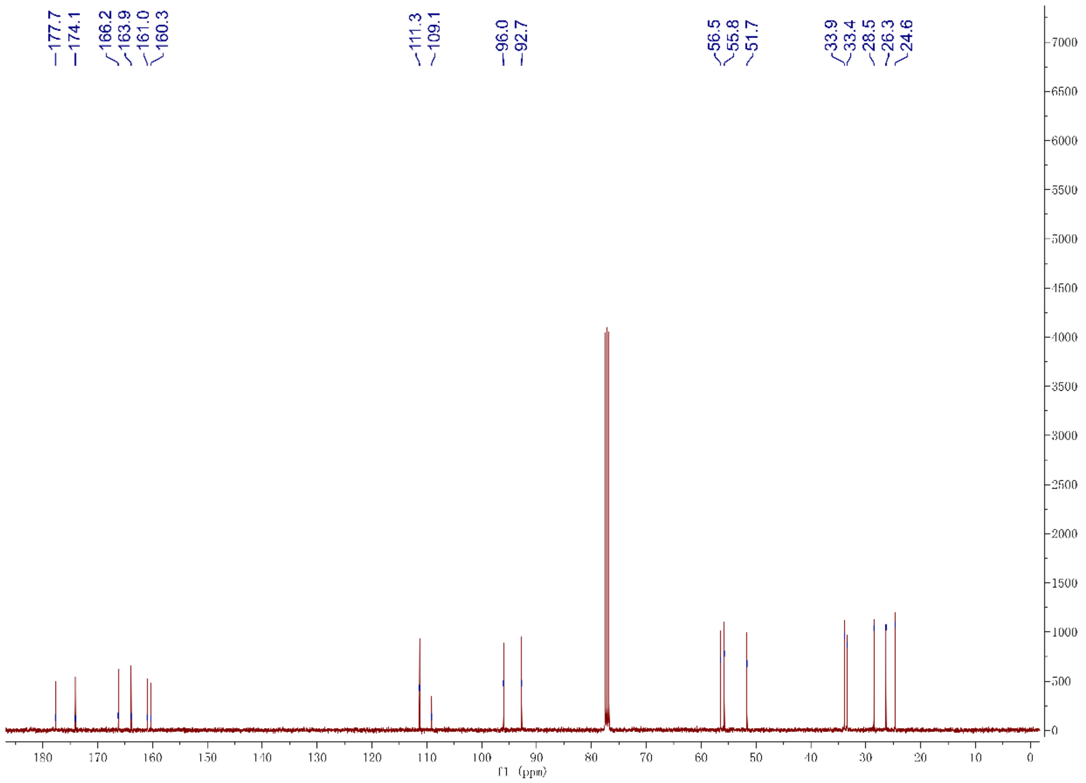

Figure S15. ^{13}C NMR (100 MHz, $CDCl_3$) spectrum of compound **7**

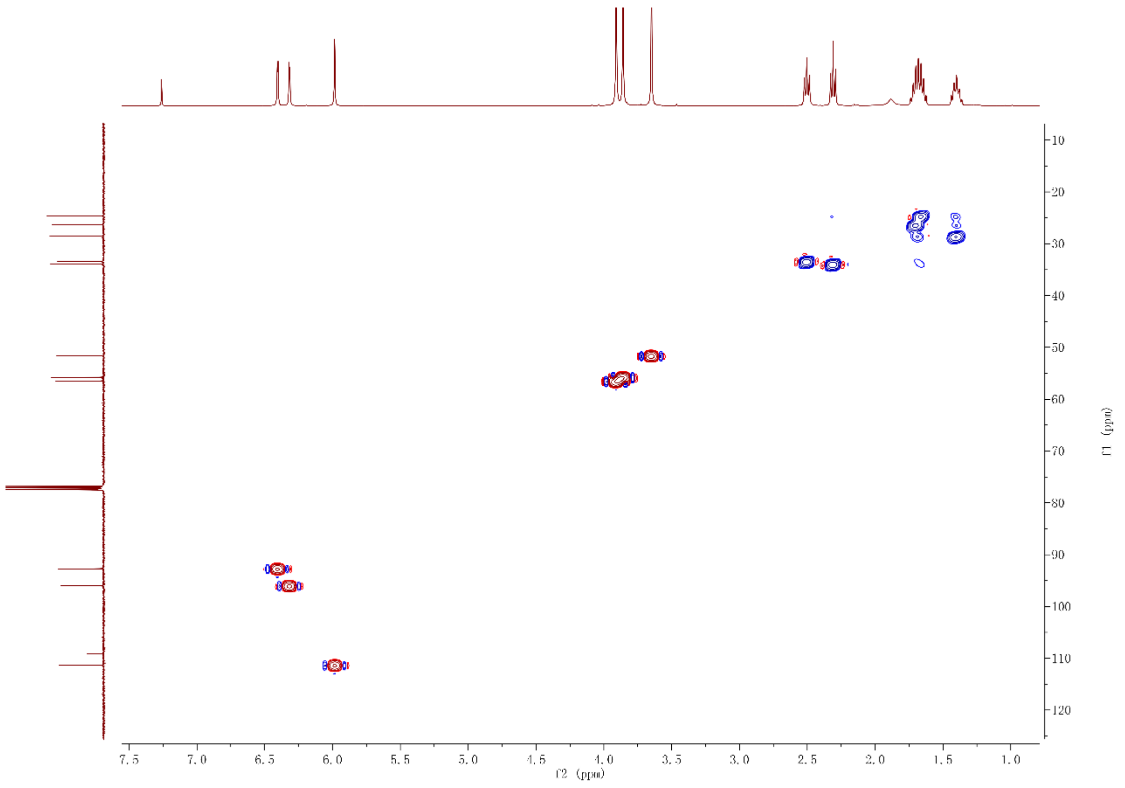

Figure S16. HMQC ($CDCl_3$) spectrum of compound **7**

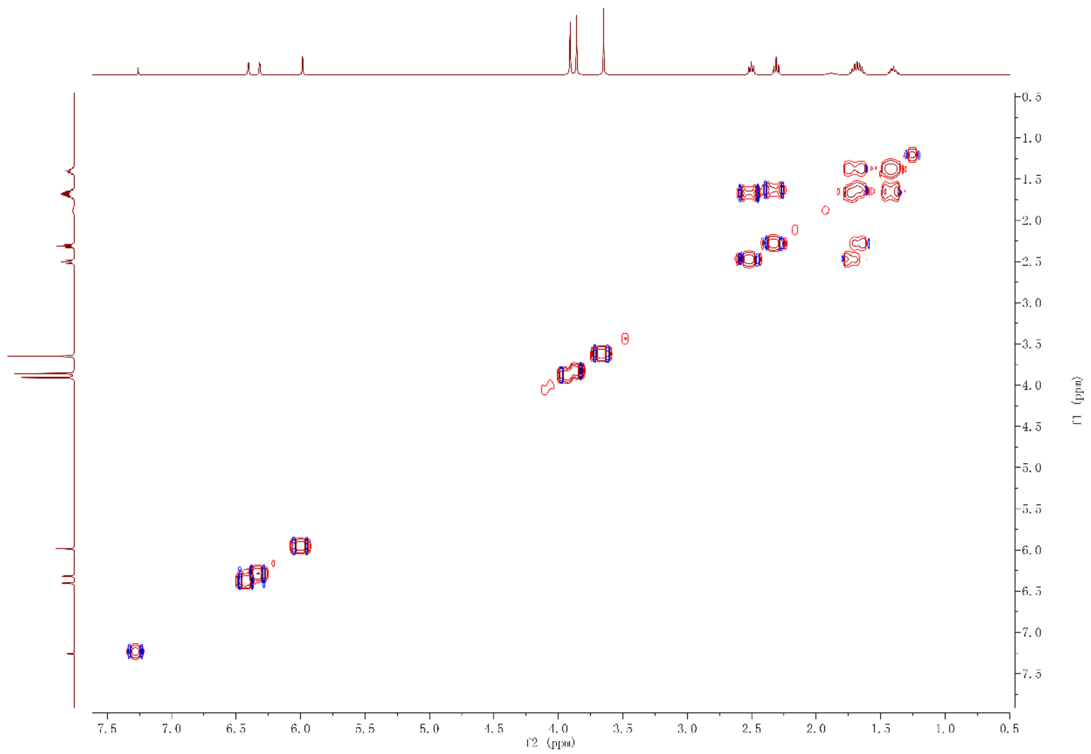

Figure S17. ^{1}H-^{1}H COSY ($CDCl_3$) spectrum of compound **7**

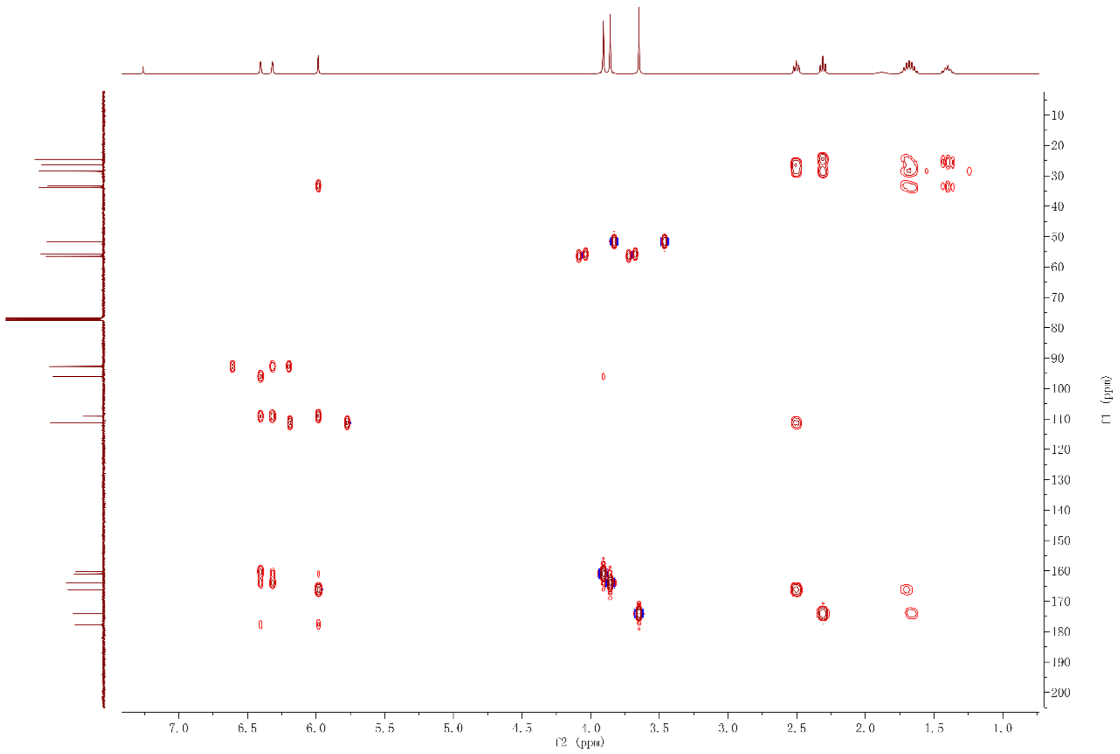

Figure S18. HMBC ($CDCl_3$) spectrum of compound **7**

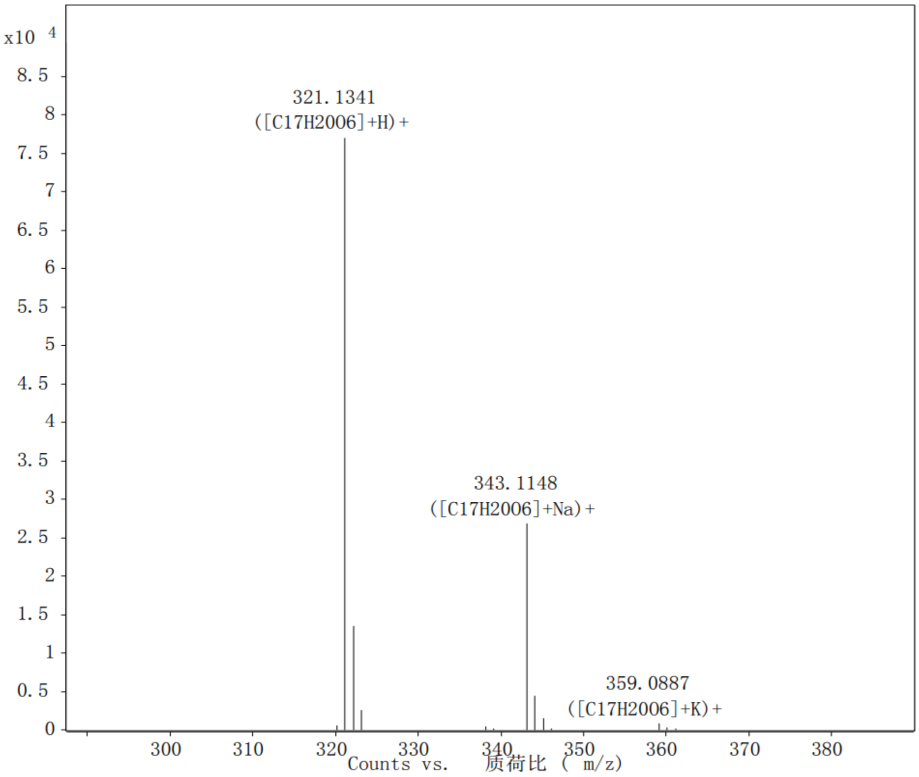

Figure S19. HR-ESI-MS spectrum of compound **8**

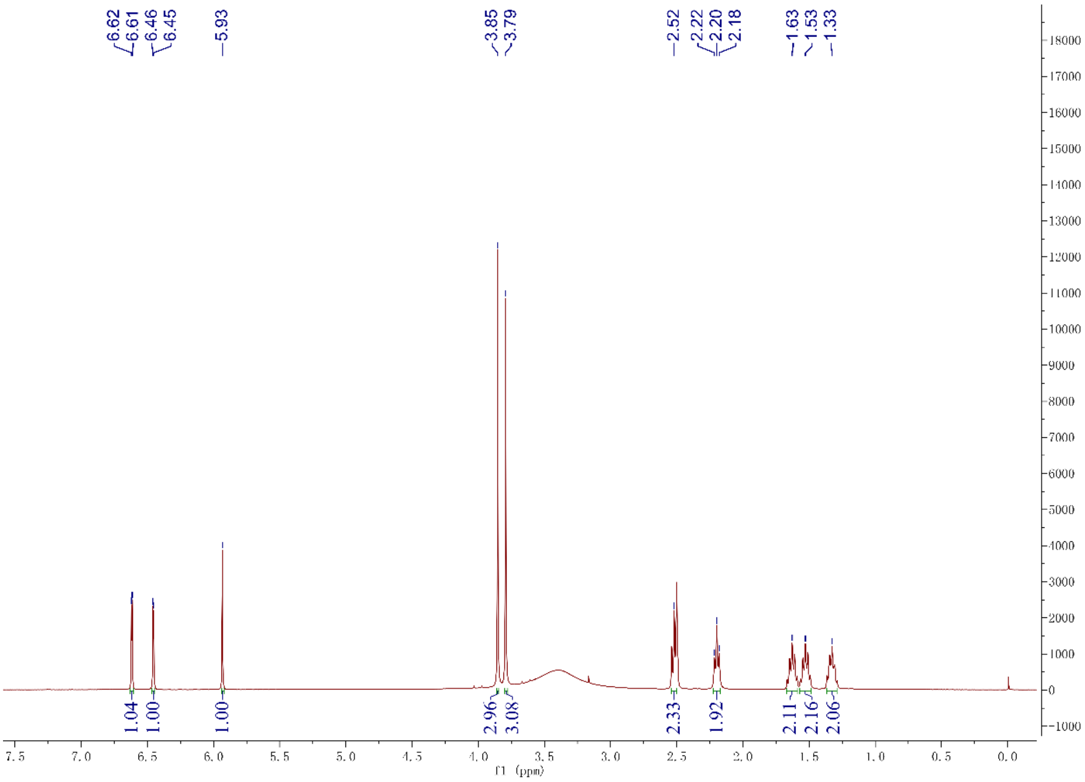

Figure S20. ^{1}H NMR (400 MHz, DMSO-d_6) spectrum of compound **8**

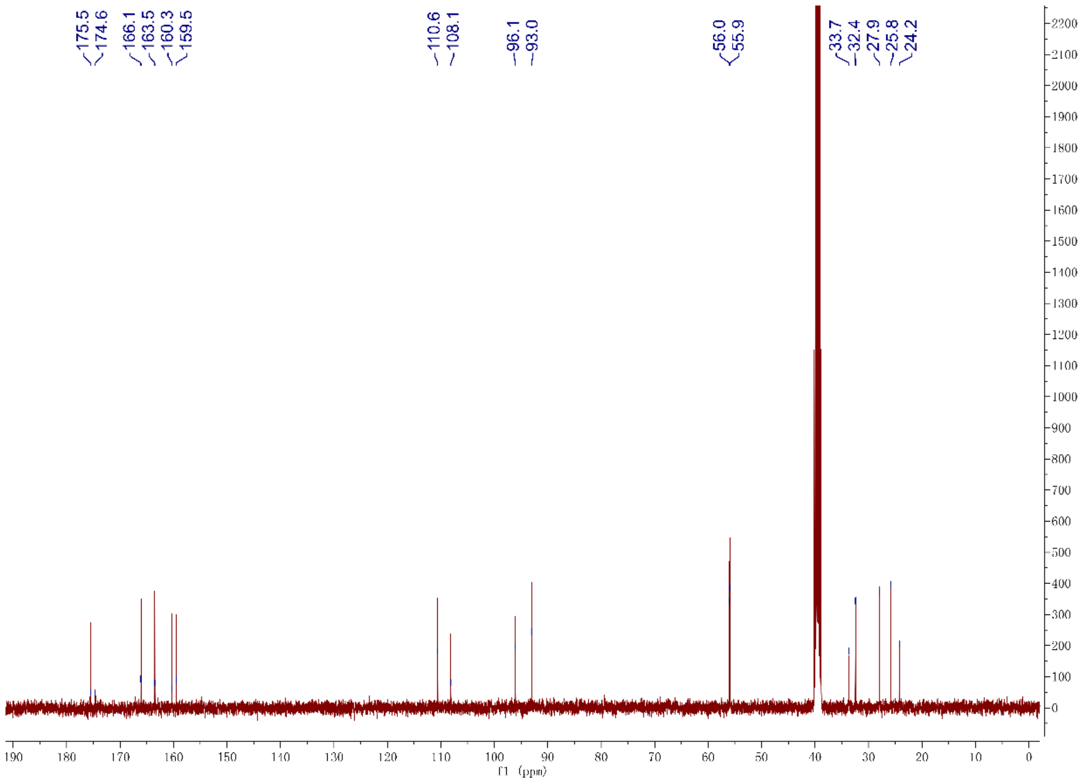

Figure S21. ^{13}C NMR (100 MHz, DMSO-d_6) spectrum of compound **8**

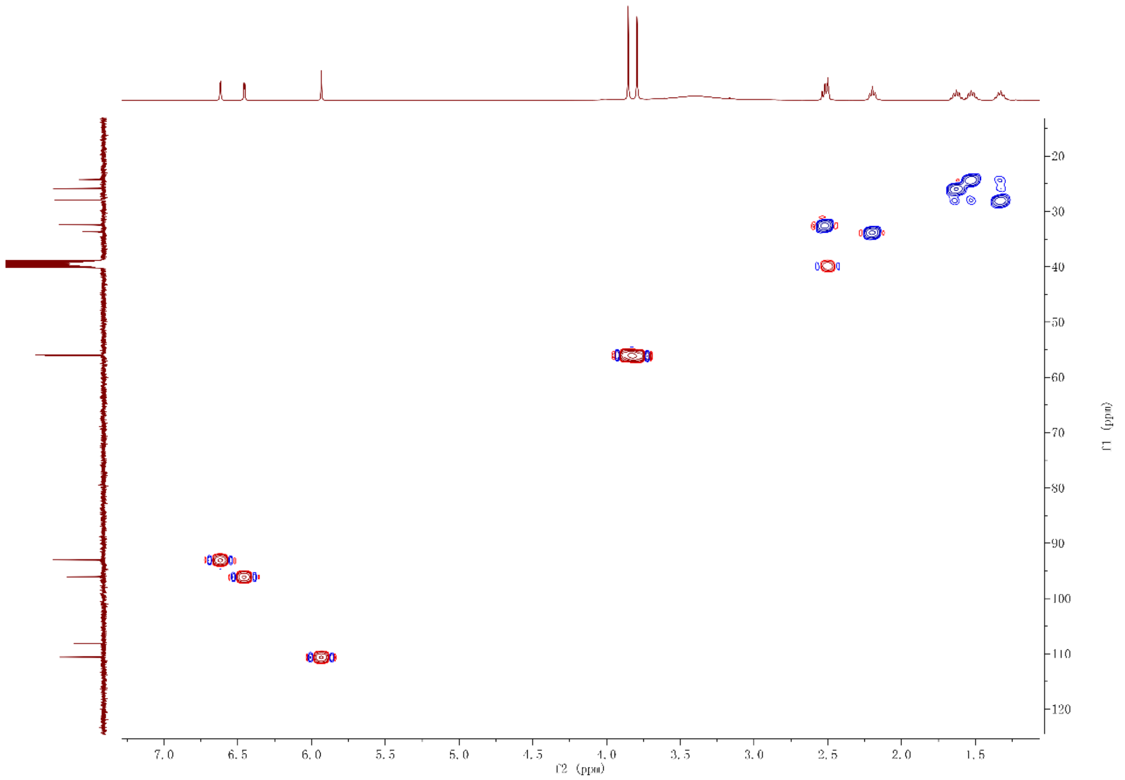

Figure S22. HMQC (DMSO-d_6) spectrum of compound **8**

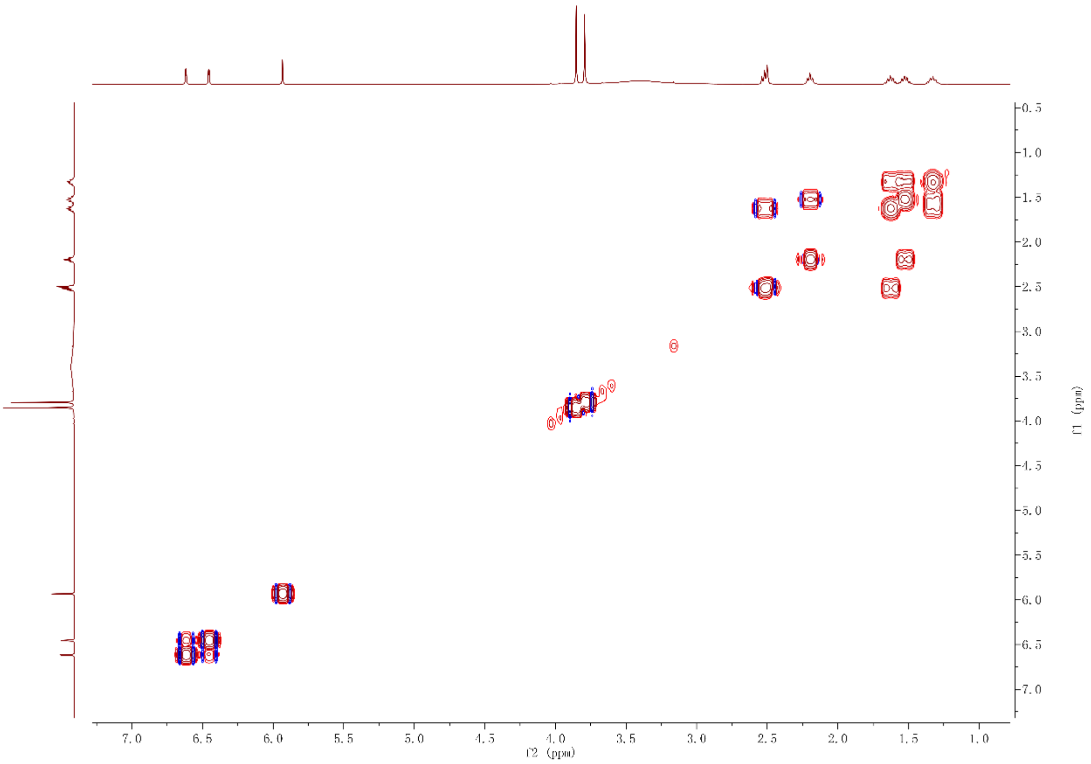

Figure S23. ^1H-^1H COSY (DMSO-d_6) spectrum of compound **8**

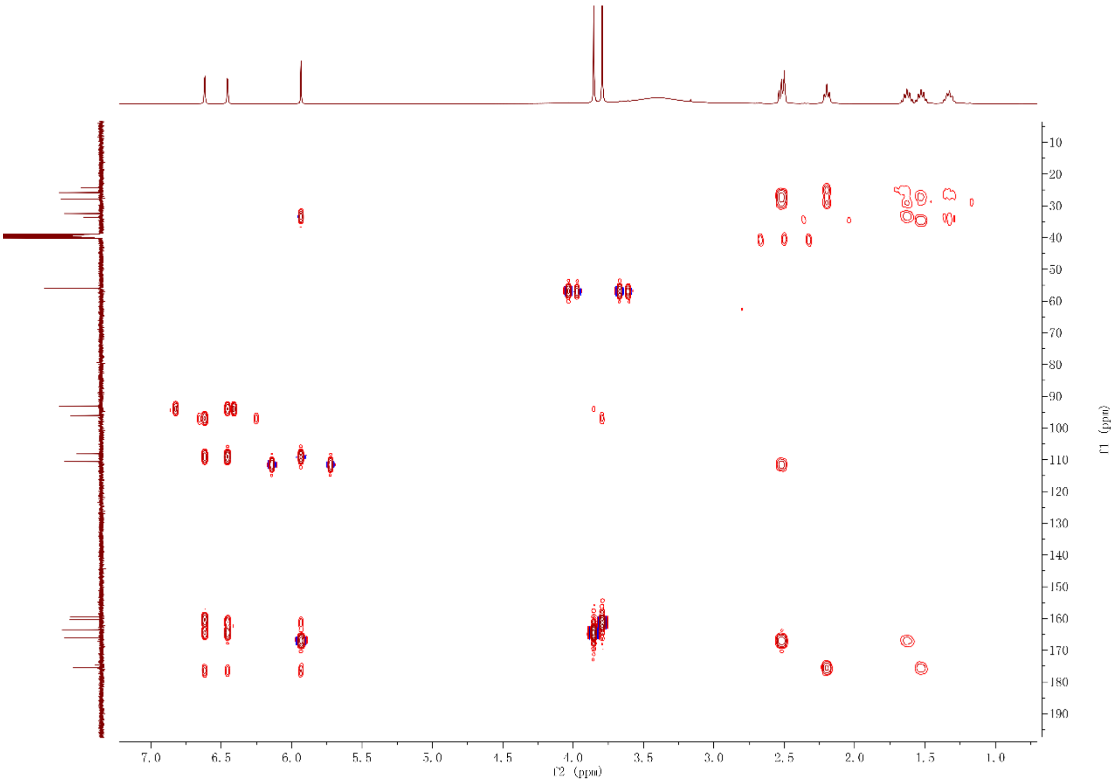

Figure S24. HMBC (DMSO-d_6) spectrum of compound **8**
